# RCN2 promotes Nasopharyngeal carcinoma progression by curbing Calcium flow and Mitochondrial apoptosis

**DOI:** 10.1007/s13402-023-00796-8

**Published:** 2023-03-23

**Authors:** Hui Yao, Siyu Zhang, Haijing Xie, Yue Fan, Mengyu Miao, Rui Zhu, Ling Yuan, Miao Gu, Yiwen You, Bo You

**Affiliations:** 1 Department of Otorhinolaryngology Head and Neck surgery, Affiliated Hospital of Nantong University, Nantong, 226001 China; 2 Institute of Otolaryngology head and neck surgery, Affiliated Hospital of Nantong University, Nantong, 226001 China; 3 Medical College of Nantong University, Nantong, 226019 China; 4 Changhai Hospital of Shanghai, No. 168 Changhai Road, Shanghai, 200433 China

**Keywords:** Nasopharyngeal carcinoma, RCN2, Mitochondrial apoptosis, Calcium flow

## Abstract

**Objective:**

Evidence suggests that calcium release from the endoplasmic reticulum (ER) can be induced to cause calcium overload, which in turn can trigger mitochondrial-dependent apoptosis. Dysregulation of systemic calcium homeostasis and changing levels of calcium-binding proteins have been shown to be associated with the malignant behavior of tumors. However, the precise molecular mechanism underlying Nasopharyngeal carcinoma (NPC) remains uncertain.

**Methods:**

Reticulocalbin (RCN2) expression in NPC was assessed using GEO database, western blot analysis and qRT-PCR. Apoptosis was assessed using flow cytometric analysis and the expression levels of apoptosis-related proteins were determined using western blot analysis. Intracellular calcium ion concentrations were measured using fluorescence imaging. The findings from these analyses were validated in vitro using nude mice models. Luciferase and ChIP assays were used to measure transcriptional regulation. Clinical significance was evaluated using tissue microarray analysis (n=150).

**Results:**

Our results showed that RCN2 promotes malignancy by causing Ca2+ flow imbalance, which leads to the initiation of the stress-mediated mitochondrial apoptosis pathway. We demonstrate that calreticulin (CALR) resides primarily in the endoplasmic reticulum and interacts with RCN2. Moreover, the transcription factors YY1 and homeobox protein goosecoid (GSC) both contribute to the initiation of RCN2 transcription by directly binding to the predicted promoter region of RCN2. Finally, high expression of RCN2 combined with high expression of GSC and YY1 may serve as an important clinical biomarker of poor prognosis in patients with NPC.

**Conclusion:**

YY1 and GSC are upstream regulators of RCN2, involved in mitochondrial calcium overload and stress-induced mitochondrial apoptosis. Thus, they can play significant role in the malignant development of NPCs.

**Supplementary Information:**

The online version contains supplementary material available at 10.1007/s13402-023-00796-8.

## Introduction

Nasopharyngeal carcinoma (NPC) is a particular malignant tumor of the head and neck, which originates from the epithelial of the nasopharyngeal mucosa with extraordinarily high incidence rates in southern China [1]. Although chemoradiotherapy has become the preferred treatment for NPC, a significant number of patients develop resistance to treatment, as well as distant metastasis and local recurrence after treatment [2]. Hence, an increased understanding of the molecular mechanisms is crucial [3–6].

The ER is the primary intracellular Ca^2+^ reservoir and plays a significant role in reversible Ca^2+^ homeostasis and dynamics [7, 8]. The sarcoplasmic/ER calcium ATPase pump (SERCA), which pumps calcium from the cytosol into the ER, is the main origin of ER calcium, and these calcium release functions are maintained by inositol 1,4,5-trisphosphate receptors (IP3Rs) and ryanodine receptor 2 (RyR2) [9–11]. The close intersection between the ER and mitochondria is called the mitochondria-associated membrane (MAM) and is composed of several molecular bridges, including IP3R, which mediates the release of Ca^2+^ from the ER to mitochondria [12]. The evidence demonstrates that abnormal intracellular calcium homeostasis via the activation or expression of SERCA pumps and IP3Rs occurs in cancer [13–15].

The ER contains many different types of proteins, such as the CREC family, which has been reported to be localized primarily in the secretory pathway of cells. Human CREC proteins are a set of multiple EF-hand Ca^2+^-bound proteins encoded by five genes: *RCN1* (encodes reticulocalbin), *RCN2* (encodes ERC-55), *RCN3* (encodes reticulocalbin-3), *SDF4* (encodes Cab45 and the splice variant Cab45-C), and *CALU* (encodes calumenin-1 and calumenin-2) [16, 17]. All members play a significant role in the Ca^2+^ regulatory process via the EF-hand [18]. In previous studies, it has been shown that reticulocalbin is involved in breast cancer infiltration owing to its involvement in the RCN1-dependent calcium ion influx [19]. Unfortunately, although RCN2 has an EF hand, it has not been previously reported whether RCN2 regulates Ca^2+^ flow. Some studies suggest that a crucial feature of the CREC family is that it assists in the transport of calcium ions in the ER, rather than the storage of calcium ions in the ER [20–22]. To date, little attention has been paid to calcium-related molecular mechanisms in the pathogenesis of the CREC family. This study attempts to thoroughly investigate whether RCN2 can mediate calcium secretion and homeostasis in NPC, as well as mitochondrial apoptosis events related to calcium flow.

Apoptosis is a programmed form of cell death, and the mitochondrial apoptosis pathway is a major mechanism involved in this process [23]. The key feature of mitochondrial apoptosis is the alteration in mitochondrial membrane permeability, which results in a disruption of mitochondrial membrane potential and the subsequent release of pro-apoptotic molecules [24]. Liu hypothesized that increased calcium transfer in the endoplasmic reticulum may lead to mitochondrial calcium overload, thereby triggering mitochondrial apoptosis [25]. Also, it is worthwhile to consider the potential involvement of RCN2, a protein that encodes multiple EF-hand Ca2+-binding proteins and has been involved in mitochondrial apoptosis [26, 27].

In the present study, first, we aimed to verify the relationship between RCN2 and the malignant behavior of NPC cells at the cellular level. Additionally, we aimed to explore whether RCN2 can regulate calcium-related phenotypes, such as mitochondrial apoptosis, and the phenomena associated with calcium. Next, we aimed to investigate the mechanism by which calcium flow is regulated by RCN2. Finally, the upregulation of RCN2 in NPC will be studied, and its effects on progression, metastasis, and prognosis will be determined.

## Materials and methods

### Cell lines and cell culture

The NPC cell lines (CNE1, CNE2, 5-8 F, 6-10B, C666–1, and TWO3) and the eternalized nasopharyngeal epithelial cell line (NP69) were gifted by Sun Yat-Sen University and Xiang-Ya School of Medicine [28]. All cells were preserved as recommended when cultured in the Affiliated Hospital of Nantong University. Cancer cells were grown in RPMI 1640 medium (cat. no. 6187–010; Gibco) containing 10% fetal bovine serum (cat. no. 16,000,044; Gibco), while NP69 was cultured in keratinocyte/serum-free medium (Invitrogen, Waltham, Massachusetts, USA) supplemented with suitable growth factors. The cells were cultured in a humidified chamber at 37 °C with 5% ambient CO_2_ [29].

### Human NPC specimens

Tissue samples were obtained from patients with pathologically confirmed NPC at the Affiliated Hospital of Nantong University with the approval of the hospital’s ethics committee (IRB number: 2020-L160). All patients provided informed consent and had not undergone any cancer treatments prior to the biopsy [30]. NPC specimens were utilized to generate a tissue microarray for in situ hybridization (ISH). Patients were followed up retrospectively for an average duration of 5.1 years.

### Western blot analysis

Cells were collected and lysed on ice using whole-cell lysate and protein concentrations were evaluated. Identical concentrations of protein were boiled, separated by SDS-PAGE, transferred to polyvinylidene fluoride membranes, and then blocked using 5% skim milk for 2 h at room temperature. The membrane was incubated overnight at 4 °C in a block with the indicated primary antibody. The antibodies used are listed in Table 1. After incubation with the corresponding secondary antibodies for approximately 1 h, the protein bands on the membranes were visualized.

**Table 1:** Antibodies used for western blot, immunohistochemistry, ISH and immunofluorescence

Antibodies	Manufacturer	Catalog numbers		Dilution (WESTERN)	Dilution (IHC)	Dilution (IF)
RCN2	proteintech		10193-2-AP	1:500	1:200	
TFAR19	proteintech		21710-1-AP			1:300
Cleaved Caspase3	CellSignaling Technology		9664	1:1000	1:500	
Cleaved Caspase12	Abcam		ab62484	1:1000	1:500	
Grp78	proteintech		11587-1-AP	1:1000	1:300	1:100
LAMP1	proteintech		55273-1-AP			1:300
MIKI67	proteintech		27309-1-AP		1:100	
Cyto-C	Abcam		ab133504		1:250	
Apaf-1	proteintech		21710-1-AP		1:200	
Caspase-9	proteintech		10380-1-AP		1:200	
Caspase-3	proteintech		19677-1-AP		1:200	
Caspase-7	proteintech		27155-1-AP		1:200	
YY1	proteintech		22156-1-AP	1:500		
GSC	Abcam		ab109024	1:1000		

### qRT- PCR

Total cellular RNA was isolated from the cells using TRIzol reagent (#15,596,026; Thermo Fisher Scientific). qRT-PCR was performed using MultiScribe Reverse Transcriptase (#4,311,235; Thermo Fisher Scientific) and SYBR Green PCR Master Mix reagents (#1,176,202 K; Thermo Fisher Scientific), following the manufacturer’s protocol. The primer sequences used in this study are listed in Table 2.

**Table 2:** The primers used for qRT-PCR experiments

Names	Sequences (5’-3’)
RCN2	Forward: 5′- CCGGACTTGTTTCTCACC − 3′
	Reverse: 5′- TGGAGCTGTCTGCCATAA − 3′
YY1	Forward: 5′-GACTGGATGGCTGGCTAGGT-3′
	Reverse: 5′-GCCTGACGTGACTTGTCTGAA-3′
GSC	Forward: 5′- GCACCGCACCATCTTCACTGAC − 3′
	Reverse: 5′- CTTAAACCAGACCTCCACTTTCTCCTC − 3′
TCF3	Forward: 5′- AGCACGAGCGTATGGGCTA-3′
	Reverse: 5′- GGGCTGAGGAGAAGGAGGAT-3′
ETS1	Forward: 5′- GAAGGATGGGCAAATCTGGTC-3′
	Reverse: 5′- GAATGGAGAAGGGAACAAAAGTGA-3′
USF1	Forward: 5′- CTTGTCCTGTGCTTGCTTAGAGT-3′ Reverse: 5′- CCAGGGAAAGGAAGAACCAATG-3′

### Transfection and transduction with siRNA, plasmids, and lentivirus

Transfection experiments were performed as previously described [29]. For the RCN2 CRISPR knockout, puromycin was designed and obtained from Genechem (Shanghai, China). Cells stably expressing Cas9 were infected with a retrovirus expressing RCN2. The corresponding sequences are listed in Table 3.

**Table 3:** Sequences of cas9 and siRNA.

Names	Sequences (5’-3’)
RCN2 cas9	Sense: CTTTGATGAGAACACTGCTC Antisense: GAGCAGTGTTCTCATCAAAG
YY1_ siRNA01	Sense: CGACGACTACATTGAACAA
	Antisense: TTGTTCAATGTAGTCGTCG
YY1_ siRNA02	Sense: CCTGAAATCTCACATCTTA
	Antisense: TAAGATGTGAGATTTCAGG
YY1_ siRNA03	Sense: GATGGTTGTAATAAGAAGT
	Antisense: ACTTCTTATTACAACCATC
GSC_ siRNA01	Sense: GCGGAGAAGTGGAACAAGA
	Antisense: TCTTGTTCCACTTCTCCGC
GSC_ siRNA02	Sense: GCACCATCTTCACTGACGA
	Antisense: TCGTCAGTGAAGATGGTGC
GSC_ siRNA03	Sense: GGCTACAACAACTACTTCT
	Antisense: AGAAGTAGTTGTTGTAGCC
TCF3_ siRNA01	Sense: CCGGATCACTCAAGCAATA Antisense: TATTGCTTGAGTGATCCGG
TCF3_ siRNA02	Sense: GAACCTGAATCCCAAAGCA Antisense: TGCTTTGGGATTCAGGTTC
TCF3_ siRNA03	Sense: AGCCTCTCTTCATCCACAT Antisense: ATGTGGATGAAGAGAGGCT
ETS1_ siRNA01	Sense: GAGCTACGATAGTTGTGAT Antisense: ATCACAACTATCGTAGCTC
ETS1_ siRNA02	Sense: GGAATTACTCACTGATAAA Antisense: TTTATCAGTGAGTAATTCC
ETS1_ siRNA03	Sense: AGGGCACCTTCAAGGACTA Antisense: TAGTCCTTGAAGGTGCCCT
USF1_ siRNA01	Sense: GCTGGACAATGACGTGCTT Antisense: AAGCACGTCATTGTCCAGC
USF1_ siRNA02	Sense: GACGACTCGGGATGAGAAA Antisense: TTTCTCATCCCGAGTCGTC
USF1_ siRNA03	Sense: CGCCGAGACAAGATCAACA Antisense: TGTTGATCTTGTCTCGGCG

### Isolation of cytoplasmic and mitochondrial proteins

Mitochondria were isolated using a mitochondria isolation kit (C3601; Beyotime Biotechnology). After infection, cells were homogenized and centrifuged for 10 min at 4 °C at 600 g for nuclei removal. For the preparation of crude mitochondria pellets, the supernatants were centrifuged again at 11,000 g for 10 min at 4 °C. Supernatants were centrifuged at 12,000 g for another 10 min to obtain cytosolic proteins. After measuring the concentration of the proteins with a BCA protein assay kit (23,225; Thermo Fisher Scientific), the proteins were used for western blotting.

### BALB/c nude mice animal models

All animal experiments were performed under a protocol for approval and acquisition from the Animal Center Laboratory of Nantong University. For tumorigenesis analysis, five-week-old male BALB/C nude mice were subcutaneously implanted with 2 × 10^6^ NPC cells with or without knockout of RCN2 to establish a xenograft tumor model. Tumor size was measured using calipers and recorded as tumor volume. After the mice were sacrificed, tumors were removed and counted using calipers and an electronic weighing machine, formalin-fixed, embedded in paraffin, and cut into sections for further study.

To investigate the effects of RCN2 metastasis in vivo, a lateral tail vein injection model was established in six-week-old male BALB/c nude mice. Briefly, 2 × 10^6^ luciferase labeled NPC cells stably transfected with knockout of RCN2 or without were injected into veins of tail (n = 6 per group) and lung metastasis were measured continually using in vivo imaging system (IVIS Spectrum III; Perkin Elmer) for seven weeks. The lungs were dissected and sliced from the mice, the sections were subjected to HE staining, and the number of lung tumor nodules was counted for further study.

### Immunohistochemistry (IHC) and in situ hybridization (ISH)

The sections were deparaffinized, rehydrated, recovered with sodium citrate, blocked with endogenous peroxidase, and incubated with primary antibody at 4 °C overnight. The primary antibodies used are listed in Table 1. The sections were subsequently washed and developed with DAB fast (3,3-diaminobenzidine) as a chromogen and counter-stained with hematoxylin. Five high-magnification microscopes at 400× magnification were selected randomly, and the staining index was divided into four scores: 0 (no staining), 1 (light brown), 2 (brown), and 3 (dark brown). The IHC staining score was calculated according to the percentage and intensity.

The ISH final scoring was obtained by the product of staining intensity and abundance, with scores of 0–9 regarded as negative expression and 10–16 regarded as high expression.

### Flow cytometry analysis

Cell apoptosis/necrosis assays were performed using an Annexin V-FITC Apoptosis Detection Kit (Beyotime, C1063). After culture, the cells were re-suspended in 100 µL of annexin binding buffer per well incubated with 5 µL of PI and 5 µL of Annexin V–FITC for 15 min in the dark. The cells were then analyzed using flow cytometry (Invitrogen).

### Calcium measurement

Mitochondrial and cytoplasmic Ca^2+^ measurements were performed using Rhod-2 AM [31] (ab142780; Abcam) and Fluo-4 AM [32] (S1060; Beyotime). Cells were incubated with 5 µM dihydro Rhod-2 AM or 5 µM Fluo-4 on glass-bottomed dishes. The cells were then washed with HBSS and images were recorded using a Leica SP8 confocal microscope at excitation 549 nm/emission 578 nm or excitation 488 nm/emission 520 nm for green and red fluorescence, respectively. Subsequently, the cells were challenged with EBSS cultured in medium containing 10% FBS. Scans were collected every 15 s for 10 min under identical conditions. The fluorescence intensity at all the time points was processed using ImageJ software, and the region of interest (ROI) was selected.

### Luciferase reporter assay

For the luciferase assay, the Dual-Luciferase Reporter Assay Kit (RG088M; Beyotime, Haimen, Jiangsu, China) was used for promoter activation assays after transfection for 24 h. Briefly, the vector was transfected into NPC cells with RCN2 cas 9/control/OE. One day post-transfection, 100 µL of Bright-Glo^™^ Luciferase Assay System was added to the incubated sample, and the bioluminescence of each sample was measured for 8 s using a luminometer (Envision) after 2 min. The sequences of primers were:

RCN2 WT-3′UTR:

5’ TTTCTCTATCGATAGGTACCTCTTTGGATTCTCATAACAC 3’(forward),

5’ GTGTTATGAGAATCCAAAGAGGTACCTATCGATAGAGAAA 3’(reverse);

RCN2 mut-3′UTR:

5’CTTAGATCGCAGATCTCGAGCTGCGATGCTCCGCGGGCGGCTAC 3’(forward),

5’GTAGCCGCCCGCGGAGCATCGCAGCTCGAGATCTGCGATCTAAG 3’(reverse);

### ChIP

The ChIP assay was performed to determine the interaction between YY1 or GSC and the RCN2 promoter using a ChIP assay kit (#27,177; Thermo Fisher Scientific), as previously described [33]. Cells were treated with 1% formaldehyde and sonicated to produce 200-1,000 bps DNA fragments. The pre-clarified supernatant was then incubated with antibodies against YY1 (5 µg) (22156-1-AP; Proteintech), GSC (5 µg) (ab109024; Abcam), or isotype control IgG (1 µg) (345,701; Millipore). PCR was performed using the following primers for RCN2 promoter-binding sites:

YY1.

forward 5′-TGATTGCACAGAAGGAATAGCTTAG-3′,

reverse 5′-CAGGTTGAGGTGGAGAAATTACATA-3′.

GSC.

forward 5′-AGAGCTCCAGGAGCCATGCT-3′,

reverse 5′-AGAAACCTCCAGCCTCTCTCATT-3′).

### Co-immunoprecipitation (Co-IP)

The Co-IP experiments were performed to determine the interaction between RCN2 and CALR using a Co-IP assay kit (#26,146; Thermo Fisher Scientific). CNE2 and 5-8 F cells were lysed in 500 µL solubilization Co-IP buffer. We use 10–50 µg of protein for positive control. Then, 30 µL of RCN2 (10193-2-AP; Proteintech, Illinois, USA) or 26 µL of CALR (PA3-900; Invitrogen) were added to the rest of the supernatant for batch binding. After the beads were collected and washed 5 times, we eluted the beads with 200 µL of spent regenerant.

### Statistical analysis

Data analysis was performed using SPSS software and GraphPad Prism (San Diego, California, USA). All quantitative data were analyzed using Student’s *t*-test and one-way ANOVA and are presented as the mean ± standard deviation (SD). Spearman’s rank correlation coefficient was used to assess the correlation between genes, and Kaplan–Meier survival curves were compared using log-rank tests. Differences between experimental conditions were considered statistically significant at P < 0.05.

## Results

### Elevated expression of RCN2 correlates with poor prognosis in NPC

**Fig. 1:**
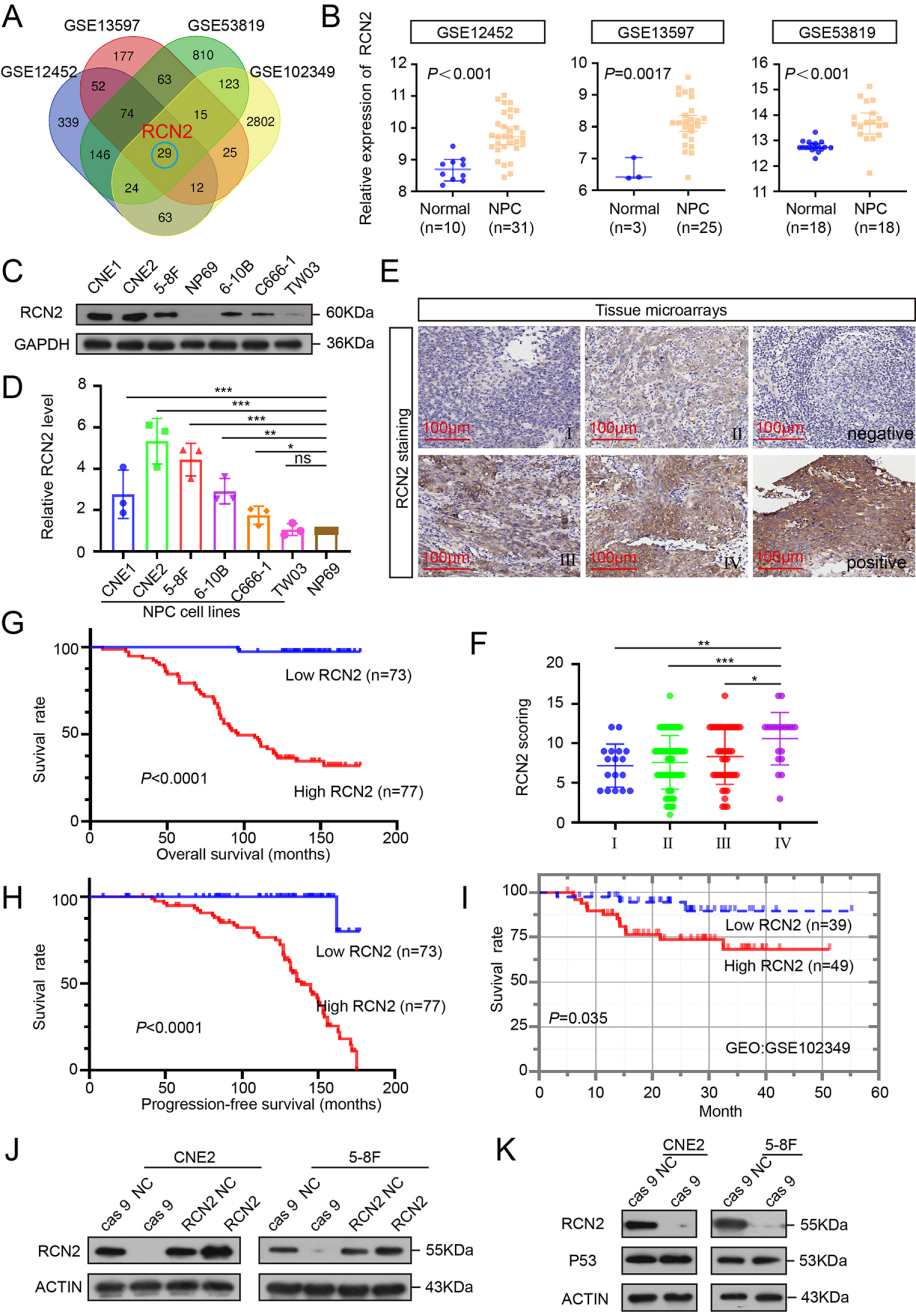
Expression and clinical significance of RCN2. **(A)** Venn diagram of upregulated genes among GEO databases (GSE12452, GSE13597, GSE53819, and GSE102349_COX). **(B)** Expression of RCN2 in three GEO databases. **(C)** RCN2 protein levels were assessed in cell lines by western blotting (WB). **(D)** qRT-PCR of RCN2 mRNA levels in NP69 and NPC cell lines analyzed using one-way ANOVA. **(E)** Representative RCN2 ISH staining (scale bar: 100 μm) and RCN2 expression in different clinical stages. **(F)** TheRCN2 staining scores were divided into low expression (scores of 0–9) or high expression (scores of 10–16). Kaplan–Meier curves representing overall survival under the influence of lymph node metastasis. **(G)** Kaplan–Meier curves representing overall survival were stratified (log-rank test). **(H)** Kaplan–Meier curves representing progression-free survival were stratified (log-rank test). **(I)** Survival differences were analyzed using the log-rank test among GSE102349_COX. **(J)** Lentivirus efficiency of RCN2 was detected by Western blot in CNE2 and 5-8 F cells. **(K)** Effects of cas 9 on p53 and RCN2 protein expressions. Data show the mean ± SD of at least three independent experiments: *P < 0.05, **P < 0.01, ***P < 0.001, ****P < 0.0001

Aberrant gene expression is repeatedly observed in NPC, which correlates with cancer progression, chemotherapy failure, and poor clinical outcomes [33, 34]. Therefore, based on the analysis of NPC microarray datasets from the Gene Expression Omnibus (GEO) databases (GSE12452, GSE13597, and GSE53819) and the survival-related dataset (GSE102349_COX), 29 significantly upregulated genes were identified. Among these, RCN2 drew our attention because of its involvement in calcium homeostasis (*P* < 0.05) (Fig. 1A, B). Analysis of TCGA Database reveals elevation of RCN2 (Supplementary Fig. 1B) and the expression of RCN2 protein in HNSC was second only to thyroid cancer among the 20 different types of tumors (Supplementary Fig. 1A). All six NPC cell lines demonstrated significantly high levels of RCN2 protein and mRNA by Western blotting and qRT-PCR (Fig. 1C, D). Then we confirmed the clinical role of RCN2 using the in-situ hybridization (ISH) data with NPC tissue. RCN2 is highly expressed in NPC tissues and high RCN2 expression were positively correlated with terminal stage disease (Fig. 1E, F). As expected from ISH results, patients with higher RCN2 expression displayed worse overall survival and progression-free survival (Fig. 1G, H). Additionally, RCN2 play a similar function in cancer progression and present similar prognostic features for NPC from GEO (GSE102349) database (Fig. 1I), and head and neck cancer (HNSC) from The Cancer Genome Atlas (TCGA) database (Supplementary Fig. 1C). Collectively, these data suggest that the overexpression of RCN2 in NPC may have significant clinical relevance.

### RCN2 regulates the NPC malignant phenotype

As the upregulation of RCN2 was significantly associated with worse survival, we investigated the direct effect of RCN2 in NPC cells. The relatively high-expression cell lines (CNE2, 5-8 F) were chosen for further study. To investigate the endogenous function of RCN2, we established RCN2 overexpression and knockout cell lines. We utilized CRISPR-Cas 9 gene editing and a lentiviral-mediated overexpression approach to develop stable RCN2 knockout and overexpression cell lines by pre-selecting single cells and transfection, respectively [35, 36]. We verified the efficiency of the RCN2 knockout via western blot. Cas 9 − 5, cas 9 − 6, and cas 9 − 7 showed high efficiency of the constructs (Supplementary Fig. 1D). The knockout efficiency of cas 9 and overexpression of CNE2 and 5-8 F were validated by qRT-PCR (Supplementary Fig. 1E). After selection, a stable knockout of the cas 9 − 7 clone was established. Knockout and overexpression efficiencies of CNE2 and 5-8 F were confirmed using Western blotting (Fig. 1J). It was observed that the negative control of cas 9 and the negative control of RCN2 had a similar effect. In addition, our findings showed that the expression of a selected group of cancer-related genes such as P53 was not significantly affected by CRISPR-cas 9. (Fig. 1K).

**Fig. 2:**
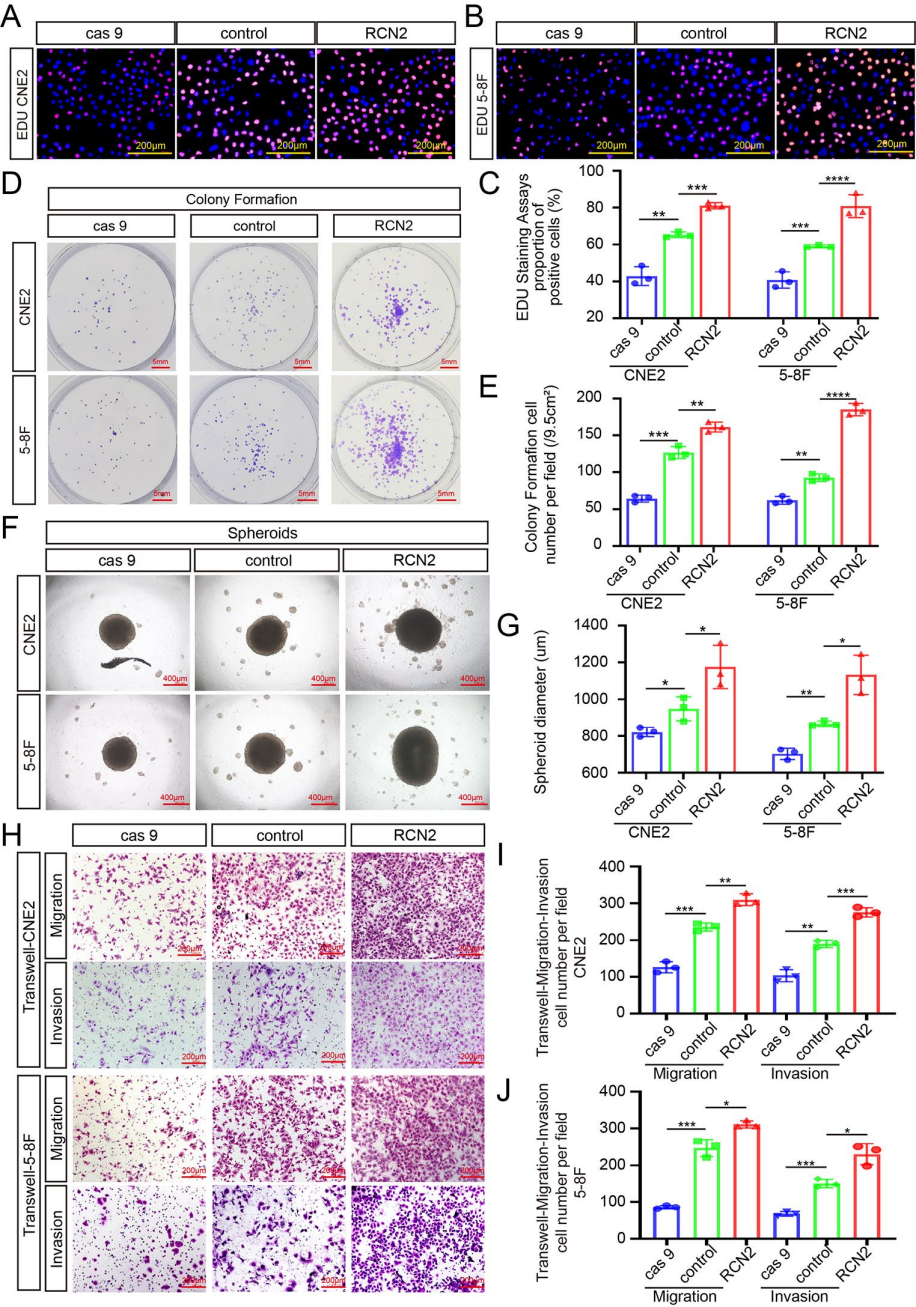
RCN2 regulates NPC malignant biological properties in NPC cells. EDU assay (scale bar: 100 μm) **(A-C)**, and colony formation assay (scale bar: 5 mm) **(D, E)** in CNE2 and 5-8 F were performed to measure cell proliferation and quantifications. **(F, G)** Cell mammosphere-forming capacity was analyzed by performing three-dimensional spheroid formation assay (scale bar: 400 μm). **(H-J)** Transwell assays (scale bar: 200 μm) were performed to analyze cell mobility and invasive capacity. Data show the mean ± SD of at least three independent experiments: *P < 0.05, **P < 0.01, ***P < 0.001, ****P < 0.0001

Next, we investigated the effects of RCN2 on the malignant biological behavior of tumor cells. CCK8, EDU, and colony formation assays were utilized to assess the cell proliferation potential, showing that the knockout of RCN2 inhibited the proliferative capacity, while cells with high RCN2 levels had a higher proliferative capacity in CNE2 and 5-8 F cells (Fig. 2A-E; Supplementary Fig. 1F). A three-dimensional sphere culture system has been shown to provide a more suitable tumor microenvironment in vivo than 2D monolayer cell culture models. Using this culture system, higher RCN2 expression was found to enhance the mammosphere-forming capacity (Fig. 2F, G). In addition, a similar tendency was observed in mobility and invasive capacity through Transwell (Fig. 2H-J) and wound-healing assays (Supplementary Fig. 1G, H). Overall, these findings suggest that RCN2 regulates the proliferation, diffusion, and spheroid formation of NPC cells in vitro.

### RCN2 regulates mitochondrial-related apoptosis driven by cellular stress in vitro

**Fig. 3:**
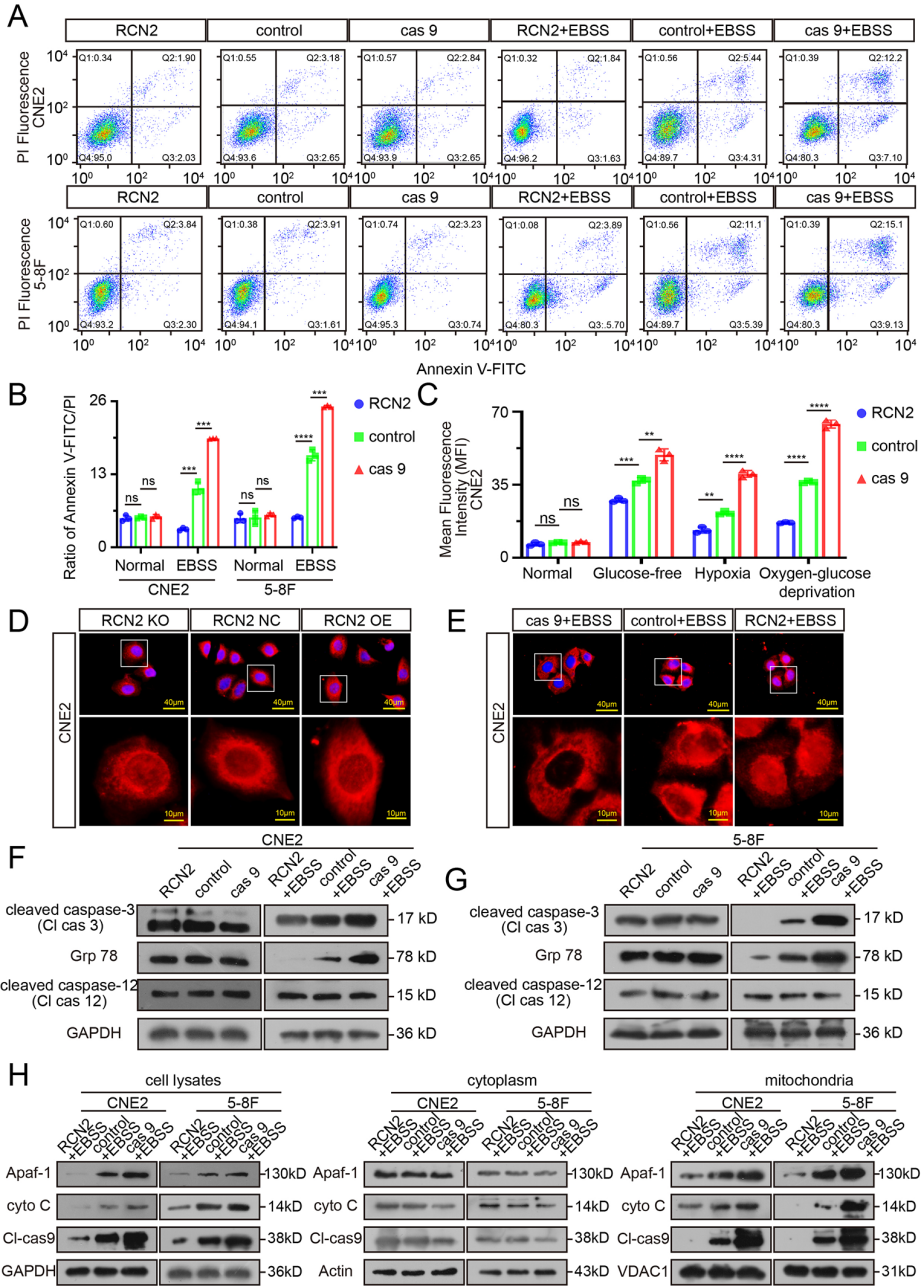
Pro-apoptotic activity driven by RCN2 under cellular stress. **(A, B)** Flow cytometry analysis of CNE2 and 5-8 F cells after transfection to induce different levels of RCN2 expression under starvation or not to measure the apoptosis rate and quantify cell apoptosis. **(C)** The ROS levels were quantified under conditions of glucose-free, hypoxia, or oxygen-glucose deprivation or not. **(D, E)** Apoptosis in CNE2 cells as defined by TFAR19 immunofluorescent staining (scale bar: 40 μm). **(F, G)** Western blot analysis of Cl-Cas 3, GRP78, and Cl-Cas 12 in CNE2 cells under starvation or not in CNE2 and 5-8 F cells. **(H)** WB analysis to assess activation of mitochondrial apoptotic pathway markers Apaf-1, cyto C, and Cl-Cas 9 under starvation or not in CNE2 and 5-8 F cells based on the separation of the cytoplasm and mitochondria. Data show the mean ± SD of at least three independent experiments: *P < 0.05, **P < 0.01, ***P < 0.001, ****P < 0.0001

Given that RCN2 is an EF-hand Ca^2+^-binding protein [37, 38], we suspected that the differential expression of RCN2 would disrupt calcium homeostasis and affect the apoptosis of NPC cells. Flow cytometry was performed to assess the extent of apoptosis. None of these differences between cells expressing different levels of RCN2 was statistically significant (Fig. 3A, B). Oxidative stress of the tumor microenvironment plays an important role in apoptotic signaling cascades. Therefore, we hypothesized that RCN2 regulates apoptosis under intracellular stress. To further validate this assumption, the above experiment was repeated in the presence of starvation-induced oxidative stress. RCN2 knockout cells comprised a significant proportion of apoptotic cells, indicating that programmed cell death was induced (Fig. 3A, B), whereas very little apoptosis developed in RCN2 overexpressed cells. Given that ROS generation is involved in stress-induced apoptosis [39, 40], we cultured cells in glucose-free, hypoxic, and oxygen-glucose deprivation medium to mediate stress. As a result, we observed that the knockout of RCN2 resulted in lower ROS levels (Fig. 3C; Supplementary Fig. 2A). In contrast, the overexpression of RCN2 significantly elevated cellular ROS levels. However, ROS did not exhibit this trend in the absence of oxidative stress (Fig. 3C; Supplementary Fig. 2A). ROS can trigger cell death via the mitochondrial apoptosis pathway [41].

Additionally, nuclear translocation of programmed cell death protein 5 (TFAR19) is an early event of apoptosis, meanwhile, caspase-3, in particular cleaved caspase-3 (the active form), is considered an apoptotic marker. Notably, the level of apoptosis in stressed cells was upregulated with the concomitant downregulation of RCN2, as detected by nuclear translocation (Fig. 3D, E) and WB (Fig. 3F, G), while RCN2 overexpressed cells showed less apoptosis. Taken together, these results indicate that RCN2 is involved in the regulation of cancer cell apoptosis under intracellular stress.

Cell death pathways are highly regulated by multiple mechanisms, including mitochondria-mediated, ER-mediated, and autophagy-dependent cell death. RCN2 was described to localize predominantly to the ER. We then examined whether RCN2 regulates cell death through endoplasmic reticulum autophagy (ER-phagy). The ER and lysosome marker molecules were stained to quantify co-localization. Unfortunately, no evidence was obtained that RCN2 influences the fusion of the ER and lysosomes under starvation conditions or not (Supplementary Fig. 2B, C). These results indicate that RCN2 does not mediate cell death via ER-phagy. The GRP78 and caspase-12 activities were evaluated to determine the level of ER stress and ER pathway apoptosis. Increased ER stress occurred in the lower RCN2 level group under starvation, whereas no difference was observed without stress (Fig. 3F, G). No significant differences were observed in cleaved caspase-12 expression of the ER-apoptotic factor (Fig. 3F, G). In summary, while our results point to a significant activation of ER stress responses under stress when RCN2 is knocked out, the absence of RCN2 does not appear to cause increases in ER pathway apoptosis.

Thus, we detected the activation of mitochondria-related intrinsic apoptotic death. Moreover, based on the separation of the cytoplasm and mitochondria, the relative content of cytochrome c (cyto C), Apaf-1, and cleaved caspase-9 protein in the cytoplasm or mitochondria was detected. The study involved the isolation and purification of mitochondria from CNE2 and 5-8 F cancer cells while retaining the cytoplasm. Protein extraction was then performed separately. The results of the study demonstrated that cyto C, Apaf-1, and cleaved caspase-9 protein was significantly increased in the cell lysates, especially in mitochondria. There was no apparent difference in the cytoplasm under stress conditions (Fig. 3H). We concluded that cell death induced by the deletion of RCN2 arose specifically from the mitochondrial apoptosis pathway rather than other cell death pathways under stress.

Taken together, our findings suggest that the deletion of RCN2 induces apoptosis in stressed cells through the activation of intrinsic mitochondrial-dependent and Apaf-1-associated pathways. High levels of RCN2 expression in a subset of cancer cells may facilitate cell survival in adverse environments.

### Confirmation of mitochondrial-related apoptosis in vivo

**Fig. 4:**
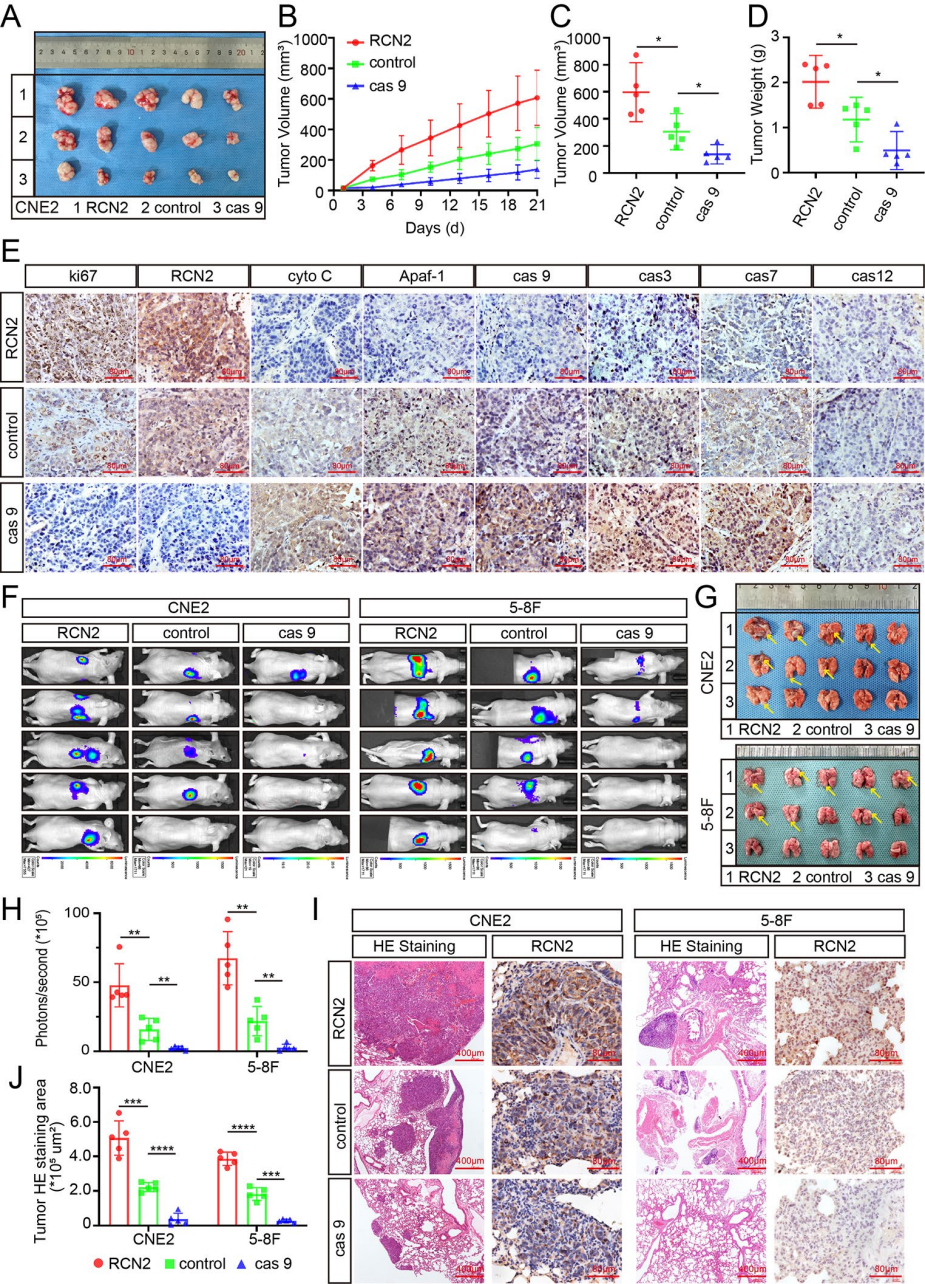
Confirmation of mitochondrial-related apoptosis in vivo. **(A)** Representative NPC xenografts in the indicated group of mice (n = 5 per group). **(B)** Tumor volume was recorded at the time points indicated. **(C)** The volume of tumors on day 21. **(D)** Tumor weight was measured after the tumors were surgically dissected. **(E)** Tumors were analyzed for MKI67, RCN2, cyto-C, Apaf-1, cas 9, cas 3, cas 7, and cas 12 expression by IHC (scale bar: 80 μm). **(F)** CNE2-luc and 5-8 F-luc cells were transfected and injected into mice through the tail vein. The lung metastasis visualization is representative of five independent experiments. **(G)** The arrow indicates the nodules of metastatic lung tumors in experimental mice. **(H)** Average intensity of fluorescence. **(I)** Typical images of histological lung metastases were analyzed using HE or RCN2 staining, and the number of lung metastatic tumors was calculated **(J)**. Data show the mean ± SD of at least three independent experiments: *P < 0.05, **P < 0.01, ***P < 0.001, ****P < 0.0001

Based on the results obtained for RCN2 in the in vitro experiments, different expression levels of RCN2 CNE2 cells were subcutaneously inoculated into nude mice, and a subcutaneous xenograft BALB/c mouse model was established to assess cell proliferation ability in vivo (Fig. 4A). After three weeks, we resected the subcutaneous tumors and measured their weights and volumes. The subcutaneous tumors in the knockout RCN2 group had a smaller volume and weighed less than those in the control group, while RCN2 overexpression was associated with a significant increase in tumor size and weight (Fig. 4A-D). IHC testing demonstrated that xenografts with lower RCN2 expression had higher levels of mitochondrial apoptosis pathway markers, such as Apaf-1, cyto-C, caspase 9, caspase 3, and caspase 7, while there was no significant difference in ER apoptosis pathway markers, such as caspase 12 (Fig. 4E).

Next, a lung metastasis model was established via tail vein injection of mice with control, RCN2 knockout, or overexpressed CNE2 and 5-8 F cells. Tumor growth and metastasis were monitored by evaluating the resulting bioluminescence values, which along with examination of lungs revealed that the RCN2 knockout group formed fewer pulmonary metastatic foci than the control group, with many more lung metastatic foci of overexpressed RCN2 cells than those of the negative control (Fig. 4F-H). Lung slices from each group were stained with hematoxylin and eosin, consistent with the in vivo imaging results (Fig. 4I, J).

In summary, these findings provide evidence that RCN2 accelerates growth and metastasis in vivo and confirm that RCN2 decreases stress-mediated apoptosis, mainly by inhibiting the mitochondrial apoptosis pathway.

### RCN2 regulates mitochondrial dysfunction and Ca^2+^ overload

**Fig. 5:**
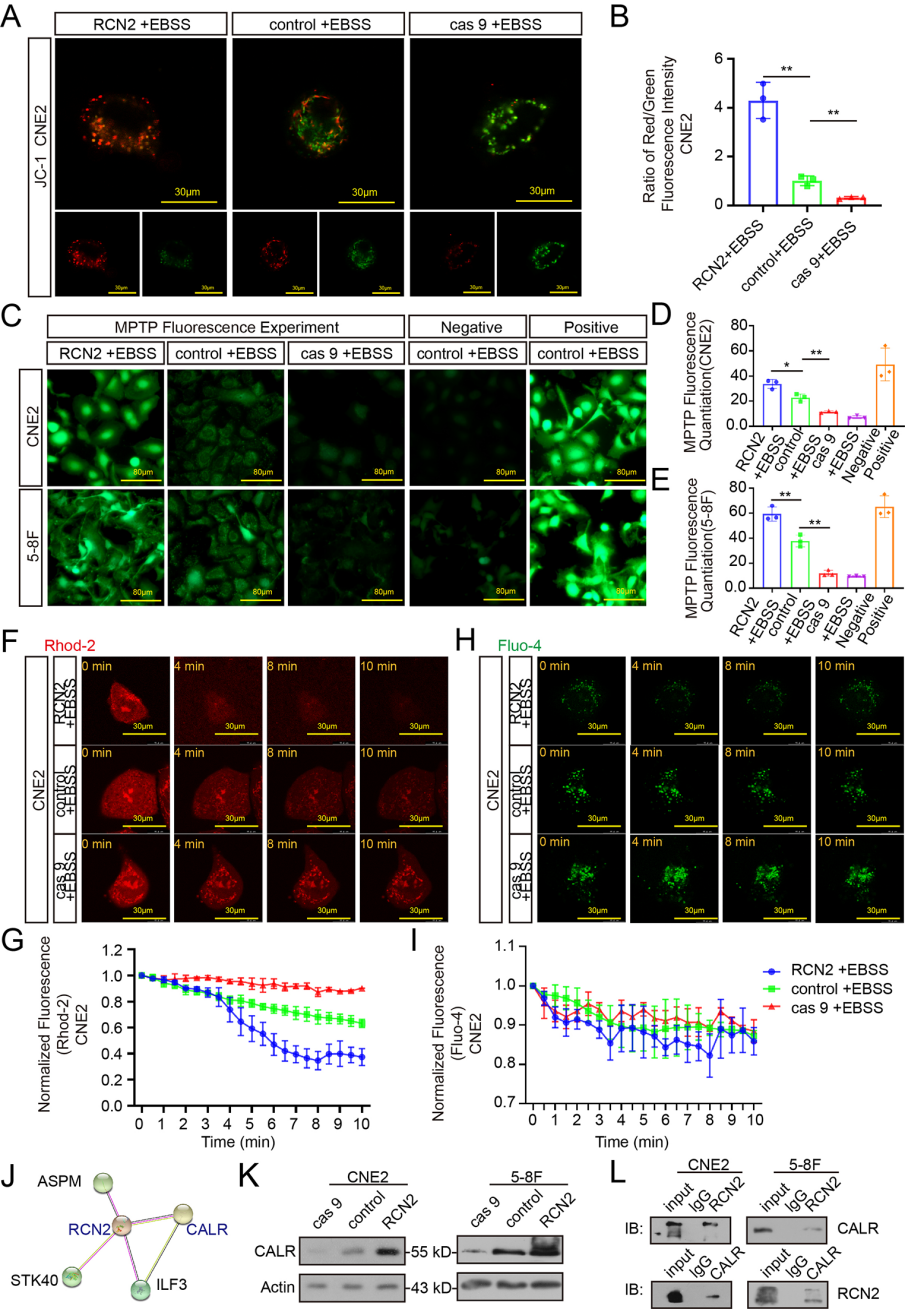
RCN2 regulates mitochondrial dysfunction and Ca^2+^ overload. **(A)** Confocal microscopy of mitochondrial membrane potential was evaluated by JC1 staining. Red puncta-maintained mitochondria; green puncta, depolarized mitochondria (scale bar: 30 μm). **(B)** Quantification of red/green represent JC1 aggregates/monomers. **(C)** MPTP openings were evaluated using the calcein cobalt method (scale bar: 80 μm) in CNE2 and 5-8 F cells. **(D, E)** MPTP openings were evaluated using the calcein cobalt metho, and the normalized relative fluorescence units (NRFU) of calcein in the experimental groups. **(F)** Mitochondrial calcium visualized by Rhod-2 under confocal microscopy (scale bar: 80 μm). **(G)** Quantitation of peak Rhod-2 fluorescence. **(H)** Cytoplasmic calcium visualized by Fluo-4 under confocal microscopy (scale bar: 80 μm). **(I)** Quantitation of peak Fluo-4 fluorescence. **(J)** RCN2 protein interaction network was generated using the STRING database. **(K)** CALR expression was detected following different expression of RCN2 by western blot in CNE2 and 5-8 F cells. **(L)** CoIP of endogenous RCN2 and CALR was performed to validate protein–protein interaction in CNE2 and 5-8 F cells. Data show the mean ± SD of at least three independent experiments: *P < 0.05, **P < 0.01, ***P < 0.001, ****P < 0.0001 MPTP: mitochondrial permeability transition pores

In addition to confirming the influence of the mitochondrial apoptotic pathway, we investigated how RCN2 regulates mitochondria-mediated apoptosis. First, JC1 staining was used to evaluate the mitochondrial membrane potential. The knockout of RCN2 cells retained monomer-emitting green fluorescence and showed mitochondrial dysfunction under cellular stress due to a reduction in mitochondrial membrane potential, while overexpressed RCN2 cells exhibited normal mitochondrial function (Fig. 5A, B; Supplementary Fig. 3A, B). Mitochondrial permeability transition pores (MPTP) also play a role in the development of mitochondrial dysfunction. Oxidative stress conditions stimulate MPTP to open, whereby part of calcein is discharged into the cytoplasm and combines with cobalt ions to lose fluorescence [42]. In our study, RCN2 knockout enhanced the opening of MPTP under oxidative stress with weaker green fluorescence, whereas RCN2 overexpression resulted in stronger green fluorescence (Fig. 5C-E). Mitochondrial calcium overload can elevate mitochondrial apoptosis by inducing permeability transitions and MPTP openings [43]. To detect cells that exhibited spontaneous calcium transients, Fluo-4 and Rhod-2 were used to measure cytosolic and mitochondrial calcium levels, respectively. As shown in the results, the knockout of RCN2 revealed increased Rhod-2 fluorescence and no difference in Fluo-4 fluorescence, which reflected an augmentation of calcium levels in the mitochondria, while calcium was gradually released from the mitochondria in the overexpressed RCN2 group under stress (Fig. 5F-I). Overall, under cellular stress damage, RCN2 deficiency stimulates MPTP opening, calcium overload in the mitochondria, mitochondrial dysfunction, cytochrome c release, and ultimately the induction of apoptosis.

RCN2 is highly connected with CALR (Calreticulin) by putative protein-protein interactions in the STRING database (Fig. 5J). CALR functions in calcium homeostasis and we found CALR expression was significantly decreased following knockout of RCN2 and increased by the overexpression of RCN2 in CNE2 and 5-8 F cell lines (Fig. 5K). In addition, co-immunoprecipitation (CoIP) assay was performed to confirm their binding (Fig. 5L). These observations demonstrate that CALR interacts with RCN2, thereby promoting calcium storage in the ER, decreasing the potential for strong signaling to mitochondria and inhibiting apoptosis.

### YY1 and GSC are upstream regulators of RCN2 and correlate with poor prognosis in HNSCC

**Fig. 6:**
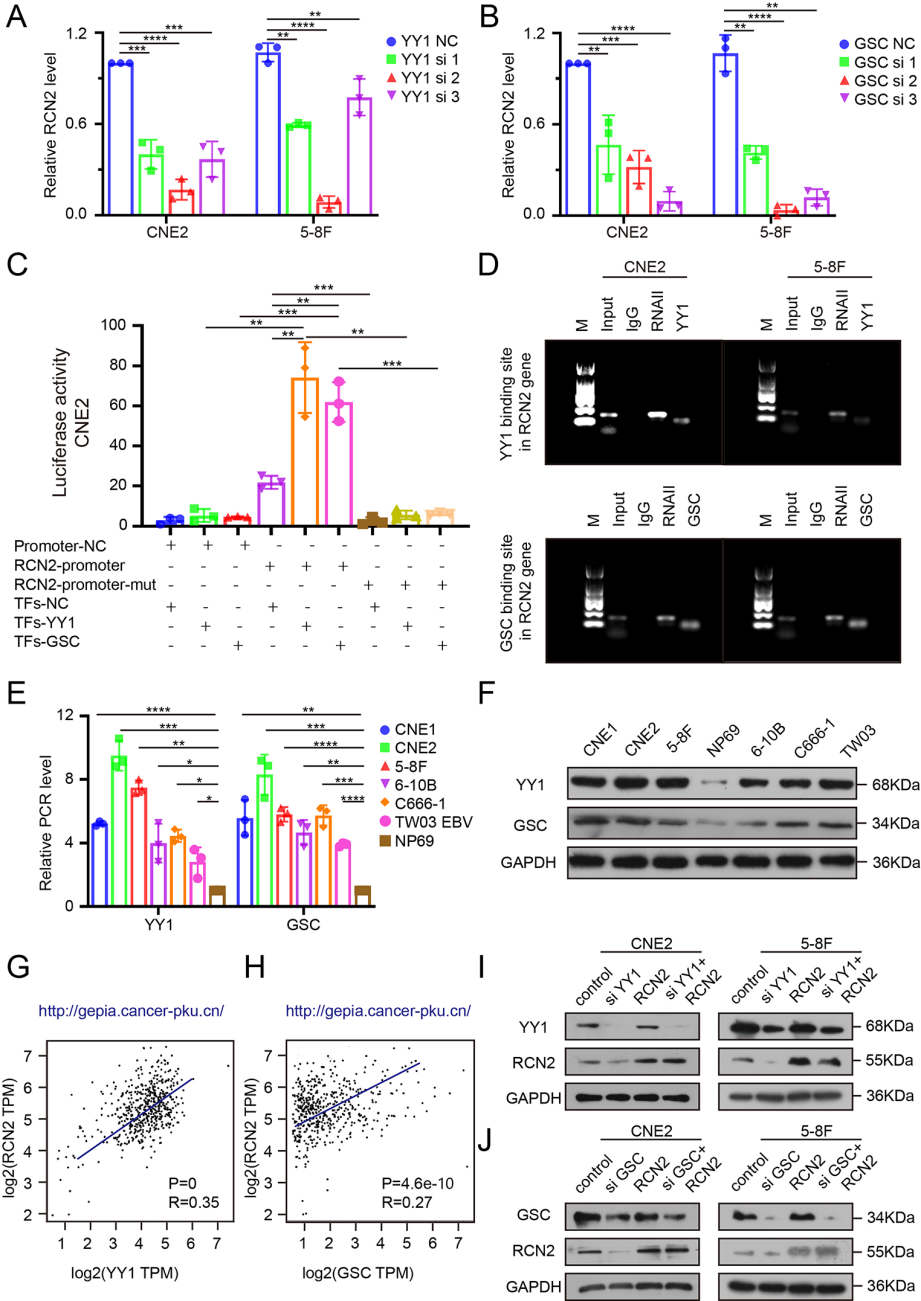
YY1 and GSC are upstream regulators of RCN2. **(A, B)** qRT-PCR analysis of RCN2 expression induced by YY1 or GSC interference transfection in CNE2 and 5-8 F cells. **(C)** Binding of YY1 or GSC to the RCN2 promoter was determined by dual-luciferase reporter assays in NPC cells. **(D)** ChIP assays in NPC cells were used to define the binding of both YY1 and GSC to the RCN2 promoter region. **(E)** qRT-PCR analysis of YY1 and GSC expression in cell lines. **(F)** Western blot (WB) analysis of YY1 and GSC in NP69 and NPC cells. **(G, H)** Analysis of the correlation between RCN2 expression level and YY1 or GSC levels in TCGA database using Spearman’s rank correlation analysis. **(I, J)** Rescue experiments were performed to verify the combination with WB of RCN2 levels. Data show the mean ± SD of at least three independent experiments: *P < 0.05, **P < 0.01, ***P < 0.001, ****P < 0.0001 ChIP: chromatin immunoprecipitation

To further elucidate the molecular mechanisms involved in the upregulation of RCN2 in NPC cells, the transcription factor binding profiles were collected from JASPER (https://jaspar.genereg.net/) [44], and the top five were selected for further analysis (YY1, GSC, ETS1, TCF3, and USF1). Silencing of YY1 or GSC was found to downregulate the expression of RCN2 (Fig. 6A, B; Supplementary Fig. 3C, D), whereas the knockdown of the other three predicted transcription factors had no influence on the expression of RCN2 (Supplementary Fig. 3E-J). We performed luciferase reporter and chromatin immunoprecipitation (ChIP) analyses to determine the promoter sites and demonstrated that RCN2 is directly transcriptionally regulated by YY1 and GSC (Fig. 6C, D). In particular, both YY1 and GSC were upregulated in NPC cell lines compared to NP69 cells by PCR and western blotting (WB) (Fig. 6E, F). In addition, YY1 or GSC expression was positively correlated with RCN2 expression in the TCGA database (Fig. 6G, H). We then verified the regulatory effects of YY1 and GSC on RCN2 expression. We observed that silencing YY1 or GSC inhibited the expression of RCN2, whereas RCN2 overexpression reversed the silencing effect of YY1 or GSC on RCN2 expression (Fig. 6I-J; Supplementary Fig. 3K, L). Hence, YY1 and GSC are the upstream transcription factors of RCN2. Moreover, patients with higher expression of YY1 and GSC had a poorer prognosis and reduced survival time, according to TCGA database (Supplementary Fig. 3M, N).

### YY1 and GSC synergistically regulate calcium flow-mediated mitochondrial apoptosis

**Fig. 7:**
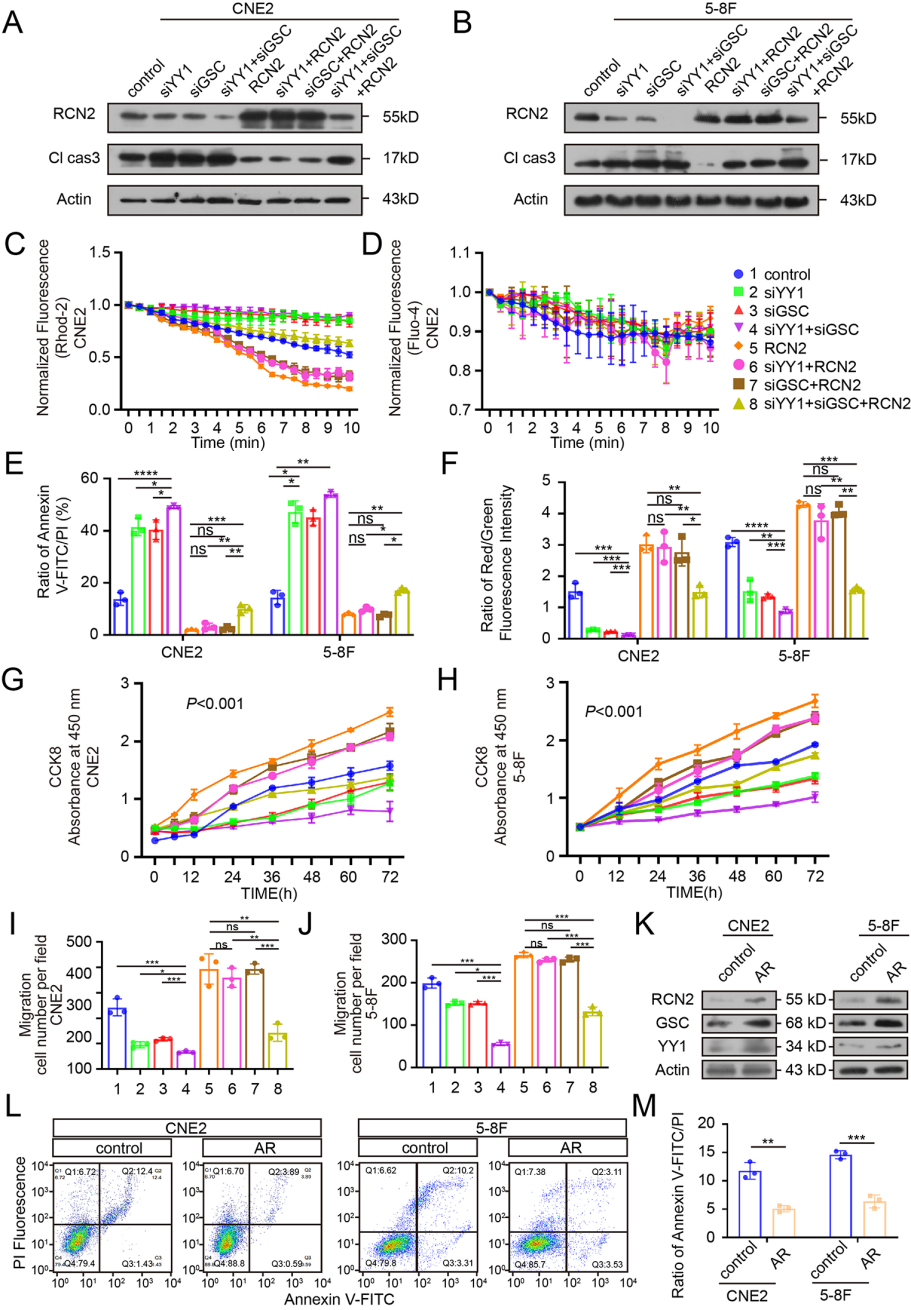
YY1 and GSC synergistically regulate calcium flow-mediated mitochondrial apoptosis. **(A, B)** Rescue experiments of knocked-down YY1 and/or GSC expression with overexpressed RCN2 or normal RCN2 to detect RCN2 and Cl-Cas3 expression by western blot (WB) analysis. **(C)** Quantitation of peak Rhod-2 fluorescence following rescue experiments to measure the levels of mitochondrial calcium. **(D)** Quantitation of peak Fluo-4 fluorescence. **(E)** Flow cytometry analysis were performed to measure the apoptosis rate and quantification of apoptosis ratio. **(F)** Rescue experiments followed by JC1 staining to measure the mitochondrial membrane potential and quantification of JC1 aggregates/monomers. CCK8 assays were carried out to measure cell proliferation upon the knockdown of YY1 and/or GSC combined with RCN2 overexpression or normal RCN2 expression in CNE2 **(G)** and 5-8 F **(H)** (two-way ANOVA). **(I, J)** Transwell assays were performed to assess migration ability in CNE2 and 5-8 F. **(K)** Western blot analysis of RCN2, YY1, GSC in CNE2 cells with anoikis resistance or not. **(L)** Flow cytometry analysis of CNE2 and 5-8 F cells after AR (Anoikis resistance) cell was induced and quantify cell apoptosis **(M)**. Data show the mean ± SD of at least three independent experiments: *P < 0.05, **P < 0.01, ***P < 0.001, ****P < 0.0001

The specific mechanism of YY1 and GSC transcriptional regulation of RCN2 is not yet fully understood. Thus, we first studied RCN2 expression after different transfections and found that the simultaneous knockdown of both YY1 and GSC resulted in a stronger downregulation of RCN2 than knockdown alone, concomitantly with a trend towards increase in cleaved caspase 3 levels, while RCN2 overexpression was unable to reverse the silencing role of YY1 and GSC simultaneously on RCN2 expression and cleaved caspase 3 levels (Fig. 7A-B; Supplementary Fig. 4A-D). These results illustrate that YY1 and GSC coordinately regulate the expression of RCN2 and apoptosis levels. Through a series of experiments, we verified that the knockdown of YY1 and/or GSC induced calcium overload in the mitochondria under stress without evident change in cytosolic calcium levels (Fig. 7C, D; Supplementary Fig. 4E, F), promoted apoptosis (Fig. 7E; Supplementary Fig. 5A), and dissipated the mitochondrial membrane potential (Fig. 7F; Supplementary Fig. 5B). Overexpressing RCN2 in the YY1 and/or GSC knockdown background reversed the mitochondrial dysfunction, and apoptosis (Fig. 7E, F; Supplementary Fig. 5A, B). Taken together, we can conclude that upstream transcription factors YY1 and GSC co-regulate mitochondrial calcium overload and mitochondrial apoptosis under stress.

We then examined whether YY1 and GSCs had co-effects on the malignant phenotype. Silencing YY1 and/or GSC decreased malignant features via decreased cell proliferation (Fig. 7G, H; Supplementary Fig. 5C, D), migration, and invasiveness (Fig. 7I, J; Supplementary Fig. 5E, F). Based on the knockdown of YY1 and/or GSC, the overexpression of RCN2 reversed the malignant phenotype.

Then, the CNE2 and 5-8 F AR (Anoikis resistance) cell was induced, screened, and obtained, and we sought to determine whether AR cells were induced successfully. RCN2, YY1 and GSC expression were compared between bulk cells and CNE2 and 5-8 F AR. Finally, we found that their expression was increased in AR cells with high metastatic potential (Fig. 7K). Then, we confirmed successful CNE2 and 5-8 F AR induction by flow cytometry (Fig. 7L, M).

Collectively, these data suggest that YY1, in synergy with GSC, is an upstream regulator of RCN2 and plays a significant role in the development of the malignant biology of NPC.

### RCN2, YY1 and GSC upregulation are associated with progression to more severe stages of NPC

**Fig. 8:**
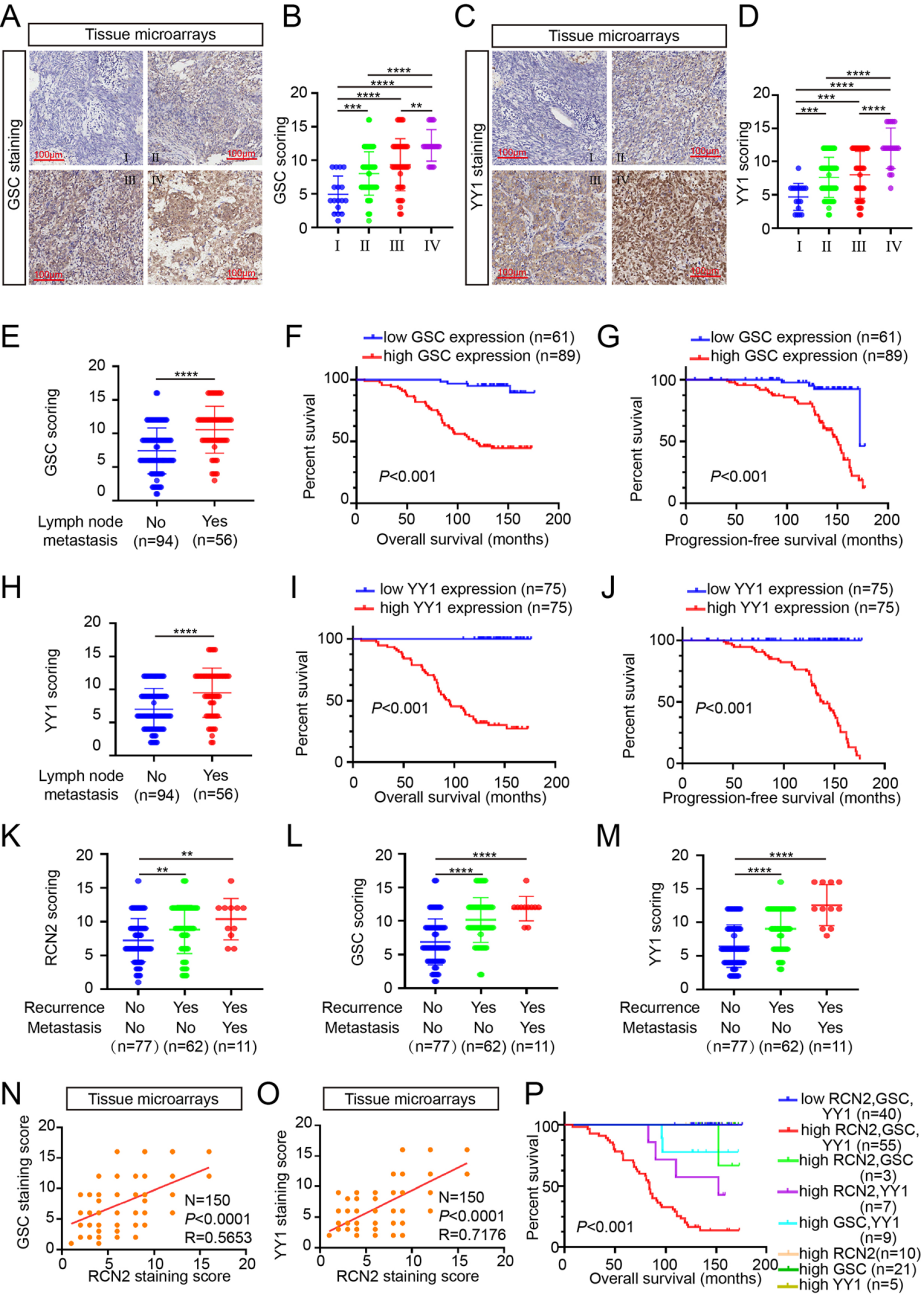
RCN2, YY1 and GSC upregulation are associated with advanced stage, recurrence, metastasis, lymph node metastasis, and poor clinical outcomes of NPC. **(A)** Representative GSC ISH staining (scale bar: 100 μm). GSC expression in different clinical stages **(B)**, as the same YY1 **(C, D)**, and lymph node metastasis **(E)**. The GSC staining scores were divided into low expression (scores of 0–9) or high expression (scores of 10–16). Kaplan–Meier curves representing overall survival **(F)** and progression-free survival **(G)** were stratified (log-rank test). The lymph node metastasis **(H)**, overall survival **(I)** and progression-free survival **(J)** of YY1 were stratified. Recurrence with or without distant metastasis in RCN2 **(K)**, GSC **(L)** and YY1 **(M)** (one-way ANOVA). **(N)** Pearson correlation between RCN2 and GSC expression was analyzed. **(O)** Pearson correlation between RCN2 and YY1 expression was analyzed. **(P)** Overall survival with differential gene expression of the three biomarkers was assessed. Data show the mean ± SD of at least three independent experiments: *P < 0.05, **P < 0.01, ***P < 0.001, ****P < 0.0001

We confirmed the clinical role of RCN2, GSC and YY1 using the ISH data with NPC tissue. High GSC, and YY1 expression were positively correlated with terminal stage disease (Fig. 8A-D). The expression of GSC correlated significantly with tumor node metastasis stages and the results showed that patients with high GSC expression had short overall or progression-free survival times (Fig. 8E-G). In addition, YY1 exhibited the same tendency (Fig. 8H-J). The results also revealed that RCN2, GSC, and YY1 were significantly correlated with NPC recurrence and distant metastasis (Fig. 8K-M). Of note, GSC or YY1 were both found to have positive protein levels correlation with RCN2 by analysis of three parallel tissue microarrays (Fig. 8N, O). We further evaluated the prognostic value and found that combined use of the three biomarkers were significantly associated with OS (Fig. 8P), and these findings suggest that high expression of RCN2 in combination with high expression of GSC and YY1 may serve as an important clinical biomarker of poor prognosis in patients with NPC.

Finally, we concluded that overexpression of RCN2, GSC, and YY1 correlate with malignancy in NPC progression and potentially constitute as prognostic biomarkers.

## Discussion

We aimed to investigate the relationship between RCN2 and the malignant behavior of NPC cells at the cellular level. Our study revealed the following findings: (i) RCN2 regulates proliferation, diffusion, and spheroid formation of NPC cells in vitro; (ii) deletion of RCN2 induces apoptosis in stressed cells; (iii) RCN2 accelerates growth and metastasis in vivo and decreases stress-mediated apoptosis; (iv) CALR interacts with RCN2, thereby promoting calcium storage in the ER; (v) silencing YY1 or GSC inhibited the expression of RCN2, whereas RCN2 overexpression reversed the silencing effect of YY1 or GSC on RCN2 expression; (vi) YY1 with GSC, is an upstream regulator of RCN2 and plays a significant role in the development of the malignant biology of NPC; (vii) Overexpression of RCN2, GSC, and YY1 correlate with malignancy in NPC progression and potentially constitute as prognostic biomarkers.

According to recent reports, no correlational studies are available on the molecular mechanisms of cellular apoptotic death in NPC under severe stress conditions. In the present study, we found that the deletion of RCN2 induces mitochondria-dependent apoptosis in stressed NPC cells, which is distinct from the existing mechanisms. More importantly, this is the first report of a molecular mechanism associated with mitochondrial-dependent apoptotic pathways activated by stress in NPC.

Importantly, Ca^2+^ signals have been shown to affect important checkpoints of the cell death process [45–47]. In their review, Giorgi reported that the pro-apoptotic transition of the mitochondria plays a key role in the regulation of cell death [48, 49]. However, the molecular nature of MPTP opening remains unresolved; there has been no comprehensive evaluation to explain the mechanism by which Ca^2+^ overload causes mitochondrial apoptosis in NPC. Our in-depth research found that CALR interacts with RCN2, thereby promotes calcium storage in the ER, inhibiting MPTP opening, calcium overload in mitochondria, mitochondrial dysfunction, cytochrome c release, and the formation of an apoptosome complex. Thus, Ca^2+^ overload causes mitochondrial apoptosis and may participate in the regulation of malignant biological behaviors in NPC.

RCN2, an EF-hand Ca^2+^-binding protein, is one of the best-characterized Ca^2+^ sensors and is mainly located in the ER [16, 17]. It is well recognized that stress signals quickly alter calcium levels, and calcium signaling is sensed and transmitted via calcium-binding proteins [50]. The mechanisms by which RCN2 induces calcium-related mitochondrial apoptosis have been intensively investigated, and we found that RCN2 combined with CALR, inhibited MPTP opening and prevented the accumulation of calcium in mitochondria, thereby impeding mitochondrial-dependent apoptosis. Furthermore, our results demonstrated that RCN2 protects NPC cells against mitochondrial apoptosis induced by various stimuli and promotes survival and proliferation. Radiotherapy and chemotherapy also induce oxidative stress, and RCN2 may help NPC survive chemoradiation-induced oxidative stress, thus contributing to CRT resistance. In a recent study, MAM (Mitochondria-associated endoplasmic reticulum membrane) is paid great attention gradually, which is endoplasmic reticulum membrane subdomain that interacts physically with mitochondria [51]. It plays an important role in the regulation of mitochondrial dynamics, calcium signaling, lipid processing and transportation, mitochondrial autophagy, and endoplasmic reticulum stress. However, we have not made a corresponding study on whether RCN2 affects ER calcium channels by regulating MAM. This is worthy of our thinking and further research. Nevertheless, this finding is significant because it emphasizes that organelles do not exist independently, and that there are molecular interactions between them. The discovery of the link between RCN2 and mitochondrial apoptosis is an important contribution to our understanding of the mechanisms of the interaction between organelles.

Yin Yang-1 (YY1) is a multifunctional transcription factor that plays a considerable regulatory role in multiple biological processes, consequently influencing the pathological processes of cancers by binding to numerous proteins and DNA [52]. Anterior studies have shown that YY1 can enhance the process of carcinoma, such as lung cancer, and repress mammary formation [52–54]. Goosecoid (GSC) is a homeobox-containing gene expressed in the spine organizer [55]. Recent studies have suggested a role for GSCs in accelerating the metastasis of human mammary tumors by triggering EMT [55, 56]. The present study reports for the first time on the high expression and oncogenic role of YY1 and GSCs in NPC. ISH demonstrated that high expression of RCN2, when combined with high expression of GSC and YY1, can significantly increase the risk of future metastases and recurrence in patients. The study utilized clinical data from 150 patients with NPC to establish the relationship between the expression levels of these three genes and patient outcomes. This study is the first to report that YY1 and GSC can co-bind to the RCN2 promoter directly and regulate its transcription in NPC cells. This regulatory mode of the two transcription factors provides a new idea for the study of molecular mechanisms in the future.

Overall, our study revealed that RCN2 is overexpressed in NPC and that RCN2 overexpression in cancerous tissues closely correlates with poor prognosis. High levels of RCN2 tightly coupled to CALR, caused MPTP to become closed, promoted calcium storage in the ER, inhibited mitochondrial-dependent apoptosis, and promoted the proliferation, migration, and invasion of NPC. Moreover, both YY1 and GSC co-regulated RCN2 transcription by directly binding to the predicted connection site of the RCN2 promoter and suppressed apoptosis mainly at the level of the mitochondria. Finally, high expression of RCN2 in combination with high expression of GSC and YY1 may serve as an important clinical biomarker of poor prognosis in patients with NPC.

In conclusion, these findings facilitate the discovery of novel mechanisms for disease treatment and provide novel insights into individualized treatment of NPC.

## Electronic supplementary material

Below is the link to the electronic supplementary material.

Supplementary Material 1

## Data Availability

Data in this study are deposited in the Omnibus (GEO) knowledge base (GEO accession numbers: GSE12452, GSE13597, GSE53819, and GSE102349_COX) and The Cancer Genome Atlas (TCGA) database.

## References

[1] T. Xiang, Y.X. Lin, W. Ma, et al., Vasculogenic mimicry formation in EBV-associated epithelial malignancies. Nat. Commun. **9**(1), 5009 (2018) 30479336 10.1038/s41467-018-07308-5 PMC6258759

[2] S-P. Chen, Q. Yang, C-J. Wang, et al., Transducin β-like 1 X-linked receptor 1 suppresses cisplatin sensitivity in nasopharyngeal carcinoma via activation of NF-κB pathway. Mol. Cancer **13**, 195 (2014) 25145705 10.1186/1476-4598-13-195 PMC4158072

[3] R. Forey, A. Barthe, M. Tittel-Elmer et al., A role for the Mre11-Rad50-Xrs2 complex in Gene expression and chromosome Organization. Mol Cell. **81**(1), 183–97.e6 (2021) 10.1016/j.molcel.2020.11.010 PMC8112817 33278361

[4] R-B. Ding, P. Chen, B.K. Rajendran et al., Molecular landscape and subtype-specific therapeutic response of nasopharyngeal carcinoma revealed by integrative pharmacogenomics. Nat. Commun **12**(1), 3046 (2021) 34031426 10.1038/s41467-021-23379-3 PMC8144567

[5] J. Sun, M. Zhang, X. Qi et al., Armadillo-repeat kinesin1 interacts with Arabidopsis atlastin RHD3 to move ER with plus-end of microtubules. Nat. Commun **11**(1), 5510 (2020) 33139737 10.1038/s41467-020-19343-2 PMC7606470

[6] N. Wang, L.D. Clark, Y. Gao et al., Mechanism of membrane-curvature generation by ER-tubule shaping proteins. Nat. Commun **12**(1), 568 (2021) 33495454 10.1038/s41467-020-20625-y PMC7835363

[7] G.E. Stutzmann, M.P. Mattson, Endoplasmic reticulum ca(2+) handling in excitable cells in health and disease. Pharmacol. Rev. **63**(3), 700–727 (2011) 21737534 10.1124/pr.110.003814 PMC3141879

[8] J. Li, C. Liu, Y. Li et al., TMCO1-mediated ca(2+) leak underlies osteoblast functions via CaMKII signaling. Nat. Commun **10**(1), 1589 (2019) 30962442 10.1038/s41467-019-09653-5 PMC6453895

[9] W. Chen, R. Wang, B. Chen et al., The ryanodine receptor store-sensing gate controls Ca2 + waves and Ca2+-triggered arrhythmias. Nat. Med. **20**(2), 184–192 (2014) 24441828 10.1038/nm.3440 PMC4269524

[10] P.A. Gorski, D.K. Ceholski, R.J. Hajjar, Altered myocardial calcium cycling and energetics in heart failure–a rational approach for disease treatment. Cell. Metab. **21**(2), 183–194 (2015) 25651173 10.1016/j.cmet.2015.01.005 PMC4338997

[11] N. Paknejad, R.K. Hite, Structural basis for the regulation of inositol trisphosphate receptors by ca(2+) and IP(3). Nat. Struct. Mol. Biol. **25**(8), 660–668 (2018) 30013099 10.1038/s41594-018-0089-6 PMC6082148

[12] M. Shin, E.R. Watson, A.S. Song et al., Structures of the human LONP1 protease reveal regulatory steps involved in protease activation. Nat. Commun **12**(1), 3239 (2021) 34050165 10.1038/s41467-021-23495-0 PMC8163871

[13] L.L. Luchsinger, A. Strikoudis, N.M. Danzl et al., Harnessing hematopoietic stem cell low intracellular calcium improves their maintenance in Vitro. Cell Stem Cell, **25**(2), 225–40.e7 (2019) 10.1016/j.stem.2019.05.002 PMC7649753 31178255

[14] X. Cen, Y. Chen, X. Xu et al., Pharmacological targeting of MCL-1 promotes mitophagy and improves disease pathologies in an Alzheimer’s disease mouse model. Nat. Commun **11**(1), 5731 (2020) 33184293 10.1038/s41467-020-19547-6 PMC7665171

[15] M. Kang, B. Tang, J. Li et al., Identification of miPEP133 as a novel tumor-suppressor microprotein encoded by miR-34a pri-miRNA. Mol. Cancer **19**(1), 143 (2020) 32928232 10.1186/s12943-020-01248-9 PMC7489042

[16] B. Honore, The rapidly expanding CREC protein family: members, localization, function, and role in disease. Bioessays **31**(3), 262–277 (2009) 19260022 10.1002/bies.200800186

[17] M. Ludvigsen, C. Jacobsen, A.B. Maunsbach et al., Identification and characterization of novel ERC-55 interacting proteins: evidence for the existence of several ERC-55 splicing variants; including the cytosolic ERC-55-C. Proteomics **9**(23), 5267–5287 (2009) 19927312 10.1002/pmic.200900321

[18] B. Honore, H. Vorum, The CREC family, a novel family of multiple EF-hand, low-affinity ca(2+)-binding proteins localised to the secretory pathway of mammalian cells. FEBS Lett. **466**(1), 11–18 (2000) 10648803 10.1016/s0014-5793(99)01780-9

[19] Z. Liu, M.G. Brattain, H. Appert, Differential display of reticulocalbin in the highly invasive cell line, MDA-MB-435, versus the poorly invasive cell line, MCF-7. Biochem. Biophys. Res. Commun. **231**(2), 283–289 (1997) 9070264 10.1006/bbrc.1997.6083

[20] F.N. Reddish, C.L. Miller, R. Gorkhali et al., Calcium Dynamics mediated by the Endoplasmic/Sarcoplasmic reticulum and related Diseases. Int J Mol Sci, **18**(5) (2017) 10.3390/ijms18051024 PMC5454937 28489021

[21] O. Batistič, M. Rehers, A. Akerman, et al. S-acylation-dependent association of the calcium sensor CBL2 with the vacuolar membrane is essential for proper abscisic acid responses. Cell. Res. **22**(7), 1155–1168 (2012) 22547024 10.1038/cr.2012.71 PMC3391015

[22] M. Ludvigsen, L. Thorlacius-Ussing, H. Vorum et al., Proteomic characterization of Colorectal Cancer cells versus normal-derived Colon mucosa cells: approaching identification of Novel Diagnostic protein biomarkers in Colorectal Cancer. Int. J. Mol. Sci **21**(10), 3466 (2020) 32422974 10.3390/ijms21103466 PMC7278953

[23] D. Zhao, X. Jiang, X. Meng et al., Low-dose Radiation reduces Doxorubicin-Induced Myocardial Injury through mitochondrial pathways. Dose Response **21**(1), 15593258231155789 (2023) 36798636 10.1177/15593258231155789 PMC9926390

[24] X. Yu, Y. Luo, L. Yang et al., P–hydroxybenzyl alcohol ameliorates neuronal cerebral ischemia–reperfusion injury by activating mitochondrial autophagy through SIRT1. Mol Med Rep, **27**(3), (2023) 10.3892/mmr.2023.12955 PMC9942263 36799156

[25] L. Ni, Y. Wei, J. Pan et al., The effects of mTOR or Vps34-mediated autophagy on methylmercury-induced neuronal apoptosis in rat cerebral cortex. Food Chem. Toxicol. **155**, 112386 (2021) 34242720 10.1016/j.fct.2021.112386

[26] W.M. Han, X.B. Hao, Y.X. Hong et al., NMDARs antagonist MK801 suppresses LPS-induced apoptosis and mitochondrial dysfunction by regulating subunits of NMDARs via the CaM/CaMKII/ERK pathway. Cell. Death Discov **9**(1), 59 (2023) 36774369 10.1038/s41420-023-01362-9 PMC9922289

[27] Y. Hu, H. Jiang, Y. Xu et al., Stomatin-like protein 2 deficiency exacerbates adverse cardiac remodeling. Cell. Death Discov **9**(1), 63 (2023) 36788223 10.1038/s41420-023-01350-z PMC9929064

[28] B. You, S. Pan, M. Gu et al., Extracellular vesicles rich in HAX1 promote angiogenesis by modulating ITGB6 translation. J. Extracell. Vesicles **11**(5), e12221 (2022) 35524442 10.1002/jev2.12221 PMC9077140

[29] L. Bao, B. You, S. Shi et al., Metastasis-associated miR-23a from nasopharyngeal carcinoma-derived exosomes mediates angiogenesis by repressing a novel target gene TSGA10. Oncogene **37**(21), 2873–2889 (2018) 29520105 10.1038/s41388-018-0183-6 PMC5966363

[30] B. You, T. Xia, M. Gu, et al. AMPK-mTOR-Mediated activation of Autophagy promotes formation of dormant polyploid Giant Cancer cells. Cancer Res. **82**(5), 846–858 (2022) 34965934 10.1158/0008-5472.CAN-21-2342 PMC9359740

[31] T. Ishii, K. Sato, T. Kakumoto et al., Light generation of intracellular ca(2+) signals by a genetically encoded protein BACCS. Nat. Commun **6**, 8021 (2015) 26282514 10.1038/ncomms9021 PMC4557345

[32] J. Ren, M. Sun, H. Zhou et al., FUNDC1 interacts with FBXL2 to govern mitochondrial integrity and cardiac function through an IP3R3-dependent manner in obesity. Sci. Adv. **6**(38), eabc8561 (2020) 32938669 10.1126/sciadv.abc8561 PMC7494344

[33] Q. Zhu, Q. Zhang, M. Gu, et al. MIR106A-5p upregulation suppresses autophagy and accelerates malignant phenotype in nasopharyngeal carcinoma. Autophagy **17**(7), 1667–1683 (2021) 32627648 10.1080/15548627.2020.1781368 PMC8354606

[34] K. Zhang, D. Liu, J. Zhao et al., Nuclear exosome HMGB3 secreted by nasopharyngeal carcinoma cells promotes tumour metastasis by inducing angiogenesis. Cell. Death Dis. **12**(6), 554 (2021) 34050127 10.1038/s41419-021-03845-y PMC8163785

[35] H.H. Hoffmann, F.J. Sánchez-Rivera, W.M. Schneider, et al., Functional interrogation of a SARS-CoV-2 host protein interactome identifies unique and shared coronavirus host factors. Cell Host Microbe, **29**(2), 267–80.e5 (2021) 10.1016/j.chom.2020.12.009 PMC7833927 33357464

[36] C. Zhao, H. Liu, T. Xiao et al., CRISPR screening of porcine sgRNA library identifies host factors associated with japanese encephalitis virus replication. Nat. Commun **11**(1), 5178 (2020) 33057066 10.1038/s41467-020-18936-1 PMC7560704

[37] I. Das, C.W. Png, I. Oancea et al., Glucocorticoids alleviate intestinal ER stress by enhancing protein folding and degradation of misfolded proteins. J. Exp. Med. **210**(6), 1201–1216 (2013) 23650437 10.1084/jem.20121268 PMC3674691

[38] Z. Zuo, R.N. Smith, Z. Chen et al., Identification of a unique ca(2+)-binding site in rat acid-sensing ion channel 3. Nat. Commun **9**(1), 2082 (2018) 29802295 10.1038/s41467-018-04424-0 PMC5970173

[39] L. Zeng, P. Gupta, Y. Chen et al., The development of anticancer ruthenium(ii) complexes: from single molecule compounds to nanomaterials. Chem. Soc. Rev. **46**(19), 5771–5804 (2017) 28654103 10.1039/c7cs00195a PMC5624840

[40] X. Liu, D.E. Cooper, A.A. Cluntun et al., Acetate production from glucose and coupling to mitochondrial metabolism in mammals. Cell, **175**(2), 502–13.e13 (2018) 10.1016/j.cell.2018.08.040 PMC6173642 30245009

[41] H.G. Lee, J.H. Yang, PKC-δ mediates TCDD-induced apoptosis of chondrocyte in ROS-dependent manner. Chemosphere **81**(8), 1039–1044 (2010) 20846705 10.1016/j.chemosphere.2010.08.045

[42] M.J. Hajipour, M. Mehrani, S.H. Abbasi et al., Nanoscale Technologies for Prevention and Treatment of Heart failure: Challenges and Opportunities. Chem. Rev. **119**(21), 11352–11390 (2019) 31490059 10.1021/acs.chemrev.8b00323 PMC7003249

[43] A. Nakamura-Ishizu, A. Ito, T. Suda, Hematopoietic stem cell metabolism during development and aging. Dev. Cell. **54**(2), 239–255 (2020) 32693057 10.1016/j.devcel.2020.06.029 PMC7418032

[44] S. Mukherjee, C. Tucker-Burden, E. Kaissi et al., CDK5 inhibition resolves PKA/cAMP-Independent activation of CREB1 Signaling in Glioma Stem cells. Cell. Rep. **23**(6), 1651–1664 (2018) 29742423 10.1016/j.celrep.2018.04.016 PMC5987254

[45] C. Giorgi, A. Romagnoli, P. Pinton et al., Ca2 + signaling, mitochondria and cell death. Curr. Mol. Med. **8**(2), 119–130 (2008) 18336292 10.2174/156652408783769571

[46] K. Zarse, S. Schmeisser, M. Groth et al., Impaired insulin/IGF1 signaling extends life span by promoting mitochondrial L-proline catabolism to induce a transient ROS signal. Cell. Metab. **15**(4), 451–465 (2012) 22482728 10.1016/j.cmet.2012.02.013 PMC4844853

[47] A.Y. Abramov, T.K. Smulders-Srinivasan, D.M. Kirby et al., Mechanism of neurodegeneration of neurons with mitochondrial DNA mutations. Brain **133**(Pt 3), 797–807 (2010) 20157008 10.1093/brain/awq015 PMC2842515

[48] G. Kroemer, J.C. Reed, Mitochondrial control of cell death. Nat. Med. **6**(5), 513–519 (2000) 10802706 10.1038/74994

[49] C. Giorgi, F. Baldassari, A. Bononi et al. Mitochondrial ca(2+) and apoptosis. Cell. Calcium **52**(1), 36–43 (2012) 22480931 10.1016/j.ceca.2012.02.008 PMC3396846

[50] L. Qiang, A. Sample, C.R. Shea et al., Autophagy gene ATG7 regulates ultraviolet radiation-induced inflammation and skin tumorigenesis. Autophagy **13**(12), 2086–2103 (2017) 28933598 10.1080/15548627.2017.1380757 PMC5788558

[51] R.A. Haeusler, T.E. Mcgraw, D. Accili, Biochemical and cellular properties of insulin receptor signalling. Nat. Rev. Mol. Cell. Biol. **19**(1), 31–44 (2018) 28974775 10.1038/nrm.2017.89 PMC5894887

[52] Z. Rong, Z. Wang, X. Wang et al., Molecular interplay between linc01134 and YY1 dictates hepatocellular carcinoma progression. J. Exp. Clin. Cancer Res. **39**(1), 61 (2020) 32272940 10.1186/s13046-020-01551-9 PMC7146959

[53] W. Xia, Y. Li, Z. Wu et al., Transcription factor YY1 mediates epithelial-mesenchymal transition through the TGFbeta signaling pathway in bladder cancer. Med. Oncol. **37**(10), 93 (2020) 32970204 10.1007/s12032-020-01414-5

[54] Y. Li, T. Li, Y. Yang et al., YY1-induced upregulation of FOXP4-AS1 and FOXP4 promote the proliferation of esophageal squamous cell carcinoma cells. Cell. Biol. Int. **44**(7), 1447–1457 (2020) 32159250 10.1002/cbin.11338

[55] T.C. Xue, N.L. Ge, L. Zhang et al., Goosecoid promotes the metastasis of hepatocellular carcinoma by modulating the epithelial-mesenchymal transition. PLoS One **9**(10), e109695 (2014) 25343336 10.1371/journal.pone.0109695 PMC4208742

[56] K.W. Kang, M.J. Lee, J.A. Song et al., Overexpression of goosecoid homeobox is associated with chemoresistance and poor prognosis in ovarian carcinoma. Oncol. Rep. **32**(1), 189–198 (2014) 24858567 10.3892/or.2014.3203

