# Supplementary material for: RCN2 promotes Nasopharyngeal carcinoma progression by curbing Calcium flow and Mitochondrial apoptosis
Source: Cell Oncol (Dordr). 2023 Mar 23;46(4):1031–48. doi: 10.1007/s13402-023-00796-8 (PMC10356900; doi:10.1007/s13402-023-00796-8)
Supplement: Supplementary file 1 — Supplementary Material 1 [file 13402_2023_796_MOESM1_ESM.docx]

**Supplementary**

**Supplementary Figure S1**

**
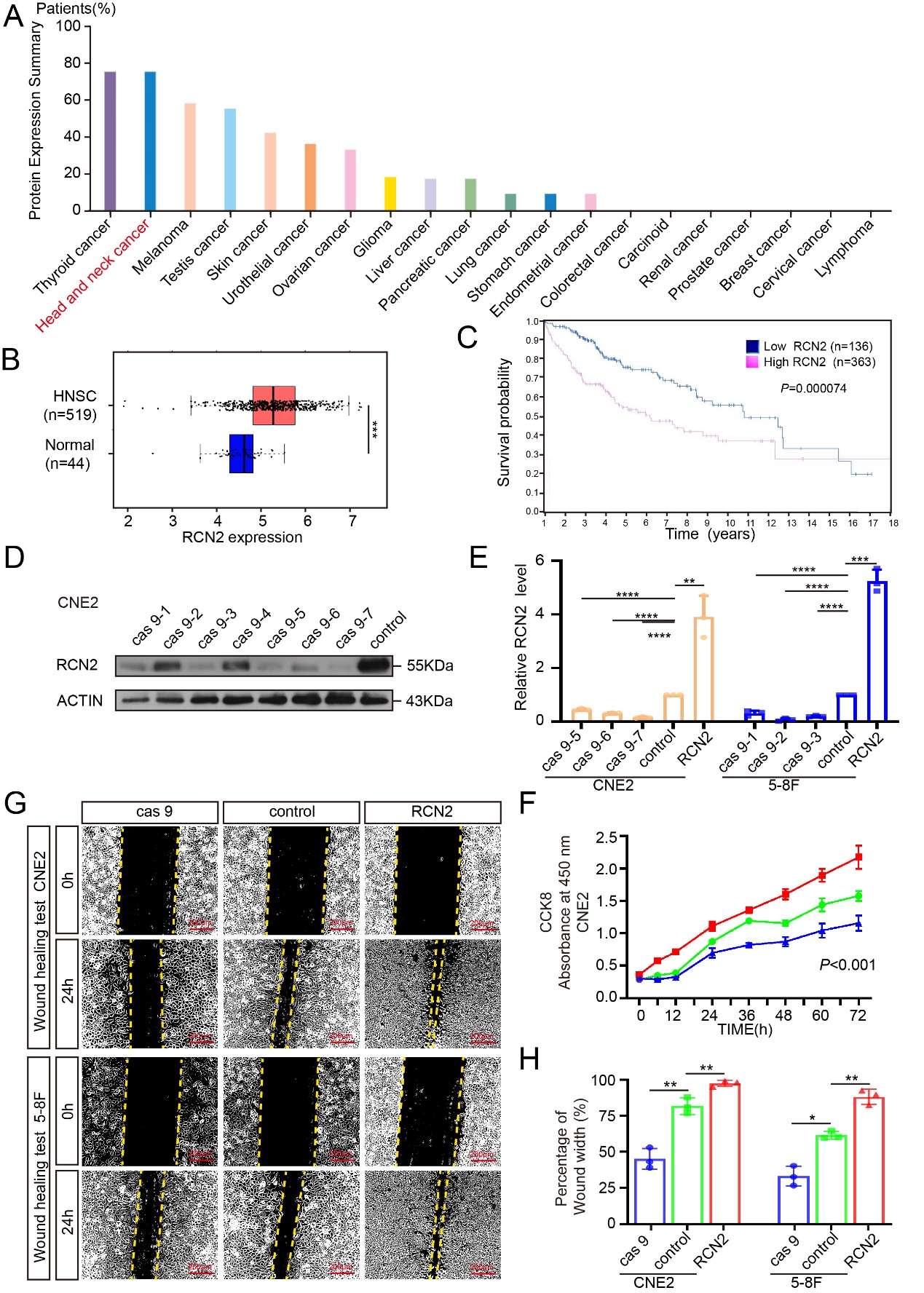
**

**Supplementary Figure S1.** RCN2 regulates NPC malignant biological properties. **(A)** Pan-cancer analysis of the protein expression summary. **(B)** RCN2 expression was evaluated in 519 patients with head and neck cancer and 44 normal controls using the TCGA database. **(C)** Kaplan–Meier analysis comparing overall survival among TCGA database. **(D)** Lentivirus efficiency of RCN2 cas 9 was detected by western blot (WB) analysis in CNE2 cells. **(E)** Lentivirus efficiency of RCN2 cas 9 KO and OE was detected by qRT-PCR and WB in CNE2 and 5-8F cells. **(F)** CCK8 was performed to measure and quantify CNE2 cell proliferation. **(G, H)** Wound-healing assay (scale bar: 200 μm) were performed to measure cell mobility and invasive capacity. Data show the mean ± SD of at least three independent experiments: **P* < 0.05, ***P* < 0.01, ****P* < 0.001, *****P* < 0.0001.

**Supplementary Figure S2**

**
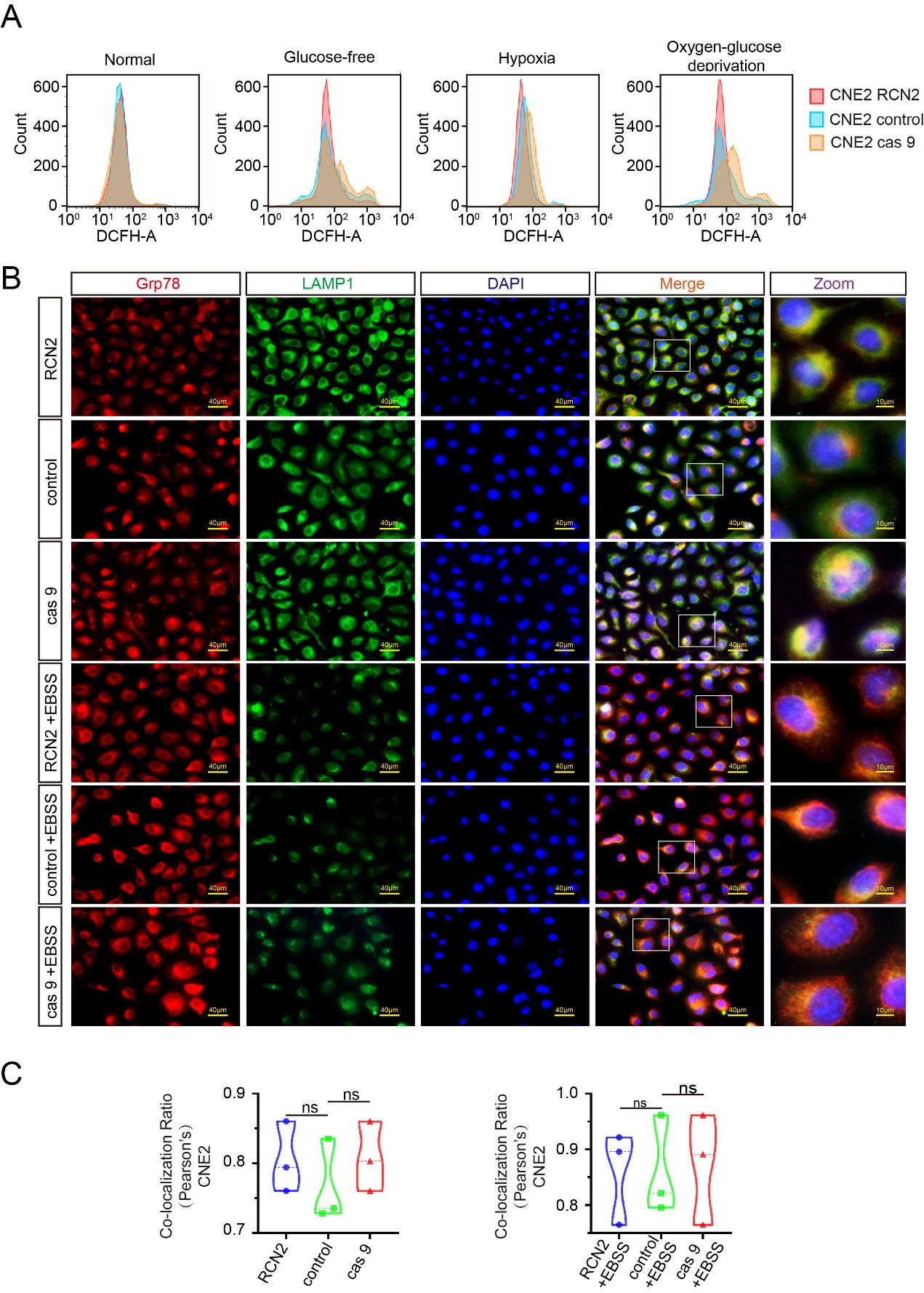
**

**Supplementary Figure S2.** Evaluation of lysosome-dependent clearing process regulation by RCN2 under cellular stress. **(A)** ROS levels were quantified by treatment with glucose-free, hypoxia, or oxygen-glucose deprivation or not. **(B)** Grp78 (red) and LAMP1 (green) co-localization was determined using immunofluorescence and confocal microscopy (scale bar: 40 μm) under starvation or not. **(C)** Quantification of Grp78 and LAMP1 co-localization by performing immunofluorescence (Student’s *t*-test). Data show the mean ± SD of at least three independent experiments.

**Supplementary Figure S3**


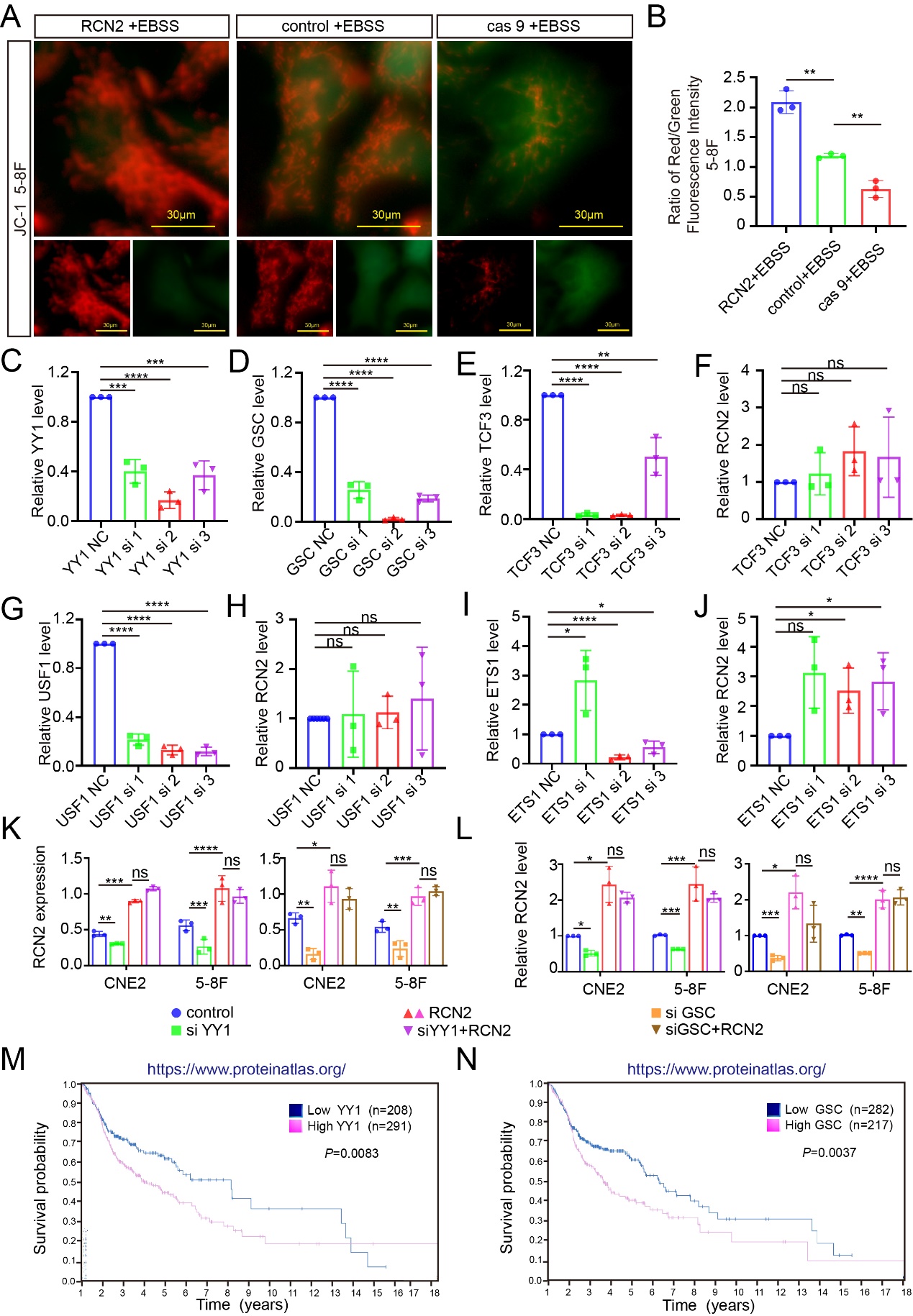


**Supplementary Figure S3.** RCN2 regulates mitochondrial dysfunction and function of RCN2 upstream regulators. **(A)** Confocal microscopy of mitochondrial membrane potential was evaluated by JC1 staining in 5-8F. Red puncta-maintained mitochondria; green puncta, depolarized mitochondria (scale bar: 30 μm). **(B)** Quantification of red/green represent JC1 aggregates/monomers. QRT-PCR analysis to determine the knockdown efficiency of YY1 **(C)**, GSC **(D)**, TCF3 **(E)**, USF1 **(G)**, and ETS1 **(I)**. qRT-PCR analysis of RCN2 expression induced by TCF3 **(F)**, USF1 **(H)**, or ETS1 **(J)** siRNA transfection. **(K, L)** Rescue experiments were performed to verify the combination with qRT-PCR analysis of RCN2 levels. **(M, N)** Kaplan–Meier analysis utilizing the log-rank test comparing overall survival among TCGA database. Data show the mean ± SD of at least three independent experiments: **P* < 0.05, ***P* < 0.01, ****P* < 0.001, *****P* < 0.0001.

**Supplementary Figure S4**


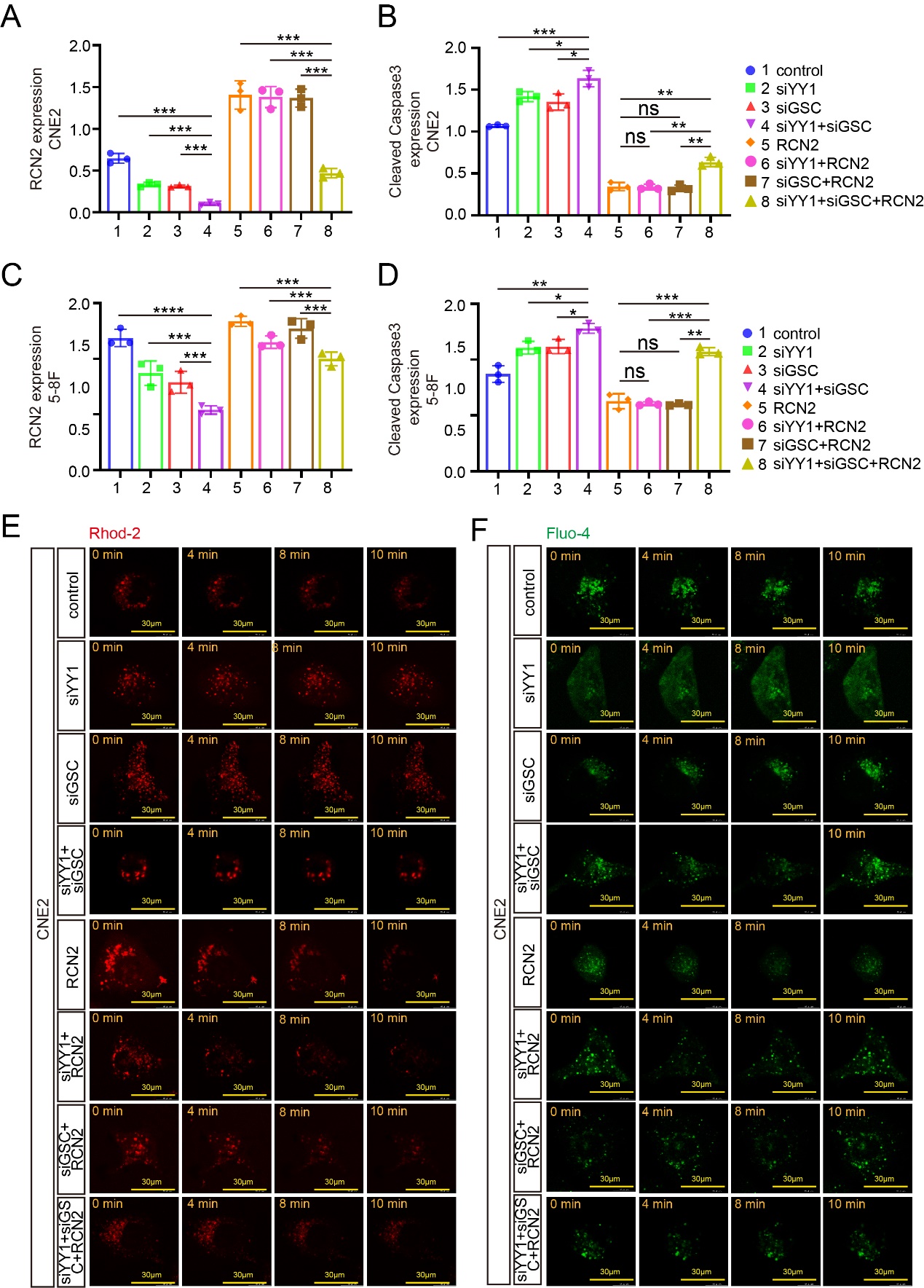


**Supplementary Figure S4.** YY1 and GSC synergistically regulate calcium flow-mediated mitochondrial apoptosis. Rescue experiments of knocked-down YY1 and/or GSC expression with overexpressed RCN2 or normal RCN2 to detect RCN2 and Cl-Cas3 expression in CNE2 **(A, B)** and 5-8F **(C, D)** by western blot analysis. **(E)** Rescue experiments of YY1 and/or GSC knock down with RCN2 overexpression or normal RCN2 expression to assess the levels of mitochondrial calcium with Rhod-2 (scale bar: 30 μm). **(F)** Rescue experiments to gauge the levels of cytoplasmic calcium with Fluo-4 (scale bar: 30 μm). Data show the mean ± SD of at least three independent experiments: **P* < 0.05, ***P* < 0.01, ****P* < 0.001, *****P* < 0.0001.

**Supplementary Figure S5**


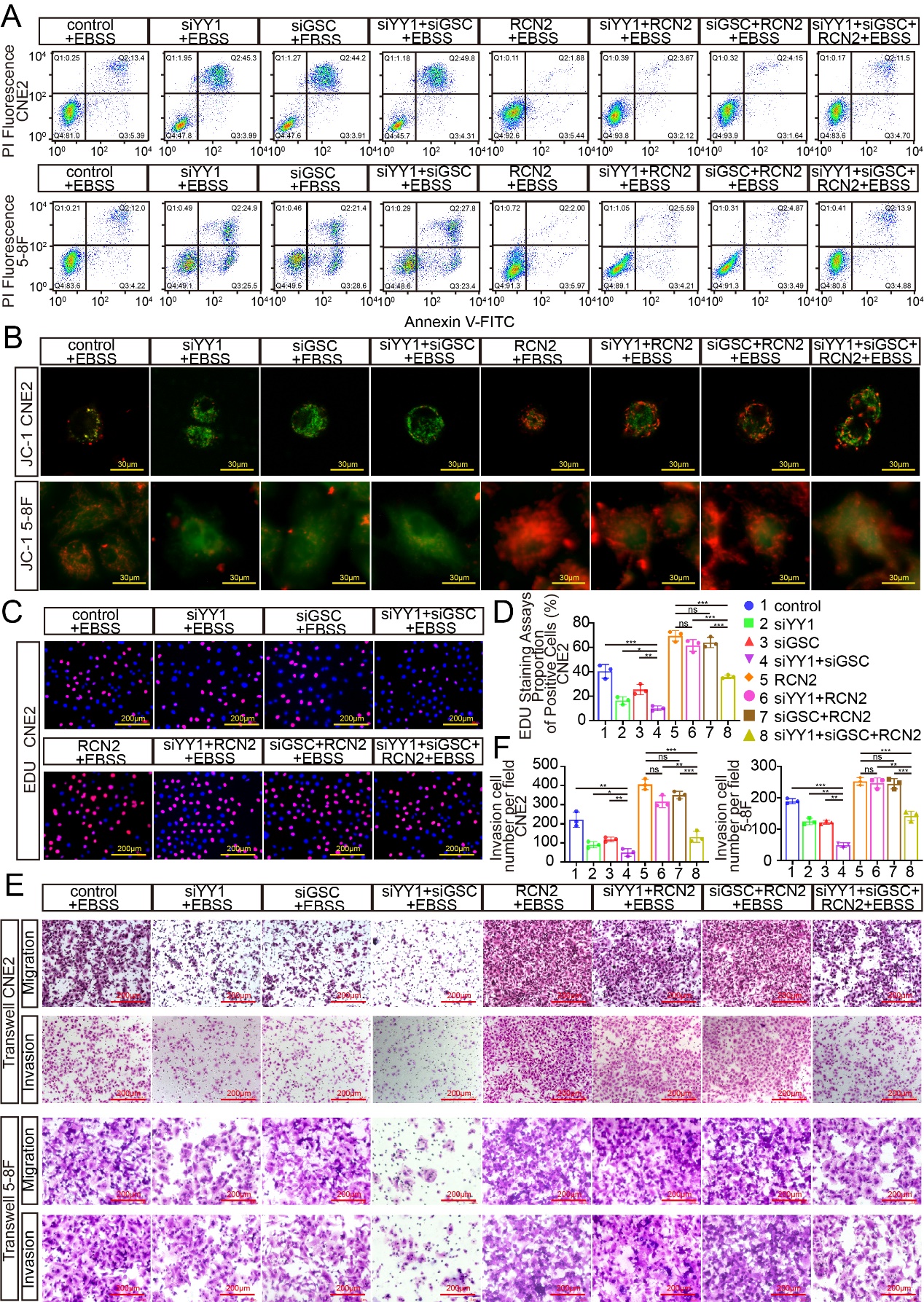


**Supplementary Figure S5.** YY1 and GSC synergistically regulate malignant phenotype. **(A)** Flow cytometry analysis were performed to measure the apoptosis rate. **(B)** Rescue experiments followed by JC1 staining to measure the mitochondrial membrane potential. **(C)** EDU assays were carried out to measure cell proliferation upon the knockdown of YY1 and/or GSC combined with RCN2 overexpression or normal RCN2 expression. **(D)** Quantitation of EDU in CNE2. **(E)** Transwell assays (scale bar: 200 μm) of CNE2 and 5-8F were performed to measure cell mobility and invasive capacity. **(F)** Quantitation of invasion in CNE2 and 5-8F. Data show the mean ± SD of at least three independent experiments: **P* < 0.05, ***P* < 0.01, ****P* < 0.001, *****P* < 0.0001.

**Supplementary Figure S6**

**
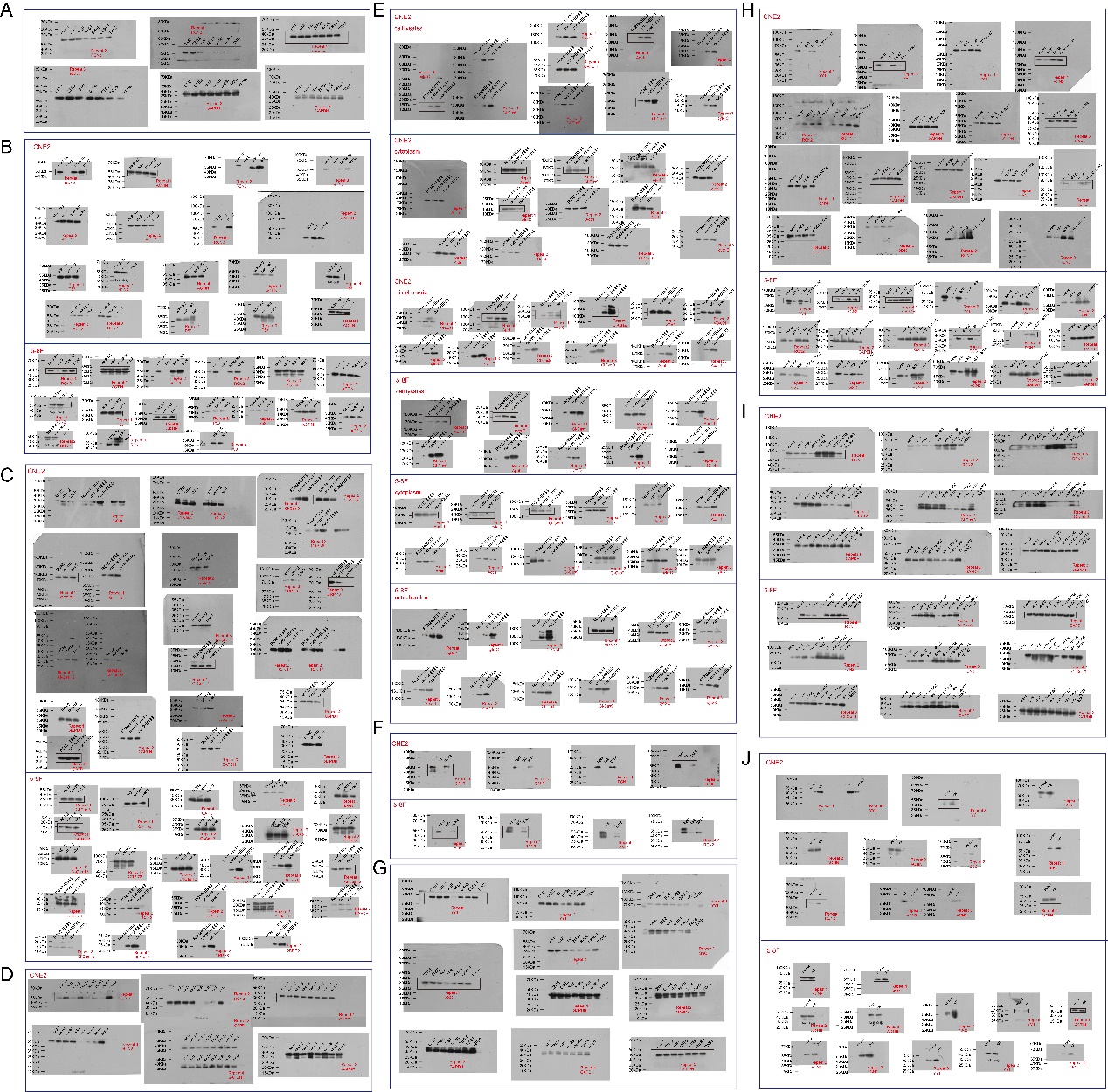
**

**Supplementary Figure S6.** Uncropped blots images for three independent experiments presented in the figures. **(A)** Immunoblots of cropped blots as shown in **Fig. 1C**. **(B)** Immunoblots of cropped blots as shown in **Fig. J, K** **(C)** Immunoblots of cropped blots as shown in **Fig. 3F, G**. **(D)** Immunoblots of cropped blots as shown in **Fig. S1D**. **(E)** Immunoblots of cropped blots as shown in **Fig. 3H**. **(F)** Immunoblots of cropped blots as shown in **Fig. 5K, L**. **(G)** Immunoblots of cropped blots as shown in **Fig. 6F**. **(H)** Immunoblots of cropped blots as shown in **Fig. 6I, J**. **(I)** Immunoblots of cropped blots as shown in **Fig. 7A, B**. **(J)** Immunoblots of cropped blots as shown in **Fig. 7K**.
